# The renoprotective efficacy and safety of genetically-engineered human bone marrow-derived mesenchymal stromal cells expressing anti-fibrotic cargo

**DOI:** 10.1186/s13287-024-03992-x

**Published:** 2024-10-23

**Authors:** Yifang Li, Alex Hunter, Miqdad M. Wakeel, Guizhi Sun, Ricky W. K. Lau, Brad R. S. Broughton, Ivan E. Oyarce Pino, Zihao Deng, Tingfang Zhang, Padma Murthi, Mark P. Del Borgo, Robert E. Widdop, Jose M. Polo, Sharon D. Ricardo, Chrishan S. Samuel

**Affiliations:** 1 Cardiovascular Disease Program, Monash Biomedicine Discovery Institute, Monash University, Clayton, VIC 3800 Australia; 2 Development and Stem Cells Program, Monash Biomedicine Discovery Institute, Monash University, Clayton, VIC 3800 Australia; 3 Department of Pharmacology, Monash University, Clayton, VIC 3800 Australia; 4 Department of Anatomy and Developmental Biology, Monash University, Clayton, VIC 3800 Australia; 5 Department of Medicine (Alfred Hospital), Central Clinical School, Monash University, Melbourne, VIC 3004 Australia; 6 Adelaide Centre for Epigenetics, School of Biomedicine, The University of Adelaide, Adelaide, SA 5005 Australia; 7 The South Australian Immunogenomics Cancer Institute, The University of Adelaide, Adelaide, SA 5005 Australia; 8 Department of Biochemistry and Pharmacology, The University of Melbourne, Parkville, VIC 3010 Australia

**Keywords:** Chronic kidney disease, Fibrosis, BM-MSCs, Relaxin, Genetic engineering

## Abstract

**Background:**

Kidney fibrosis is a hallmark of chronic kidney disease (CKD) and compromises the viability of transplanted human bone marrow-derived mesenchymal stromal cells (BM-MSCs). Hence, BM-MSCs were genetically-engineered to express the anti-fibrotic and renoprotective hormone, human relaxin-2 (RLX) and green fluorescent protein (BM-MSCs-eRLX + GFP), which enabled BM-MSCs-eRLX + GFP delivery via a single intravenous injection.

**Methods:**

BM-MSCs were lentiviral-transduced with human relaxin-2 cDNA and GFP, under a eukaryotic translation elongation factor-1α promoter (BM-MSCs-eRLX + GFP) or GFP alone (BM-MSCs-eGFP). The ability of BM-MSCs-eRLX + GFP to differentiate, proliferate, migrate, produce RLX and cytokines was evaluated in vitro, whilst BM-MSC-eRLX + GFP vs BM-MSCs-eGFP homing to the injured kidney and renoprotective effects were evaluated in preclinical models of ischemia reperfusion injury (IRI) and high salt (HS)-induced hypertensive CKD in vivo. The long-term safety of BM-MSCs-RLX + GFP was also determined 9-months after treatment cessation in vivo.

**Results:**

When cultured for 3- or 7-days in vitro, 1 × 10^6^ BM-MSCs-eRLX + GFP produced therapeutic RLX levels, and secreted an enhanced but finely-tuned cytokine profile without compromising their proliferation or differentiation capacity compared to naïve BM-MSCs. BM-MSCs-eRLX + GFP were identified in the kidney 2-weeks post-administration and retained the therapeutic effects of RLX in vivo. 1–2 × 10^6^ BM-MSCs-eRLX + GFP attenuated the IRI- or therapeutically abrogated the HS-induced tubular epithelial damage and interstitial fibrosis, and significantly reduced the HS-induced hypertension, glomerulosclerosis and proteinuria. This was to an equivalent extent as RLX and BM-MSCs administered separately but to a broader extent than BM-MSCs-eGFP or the angiotensin-converting enzyme inhibitor, perindopril. Additionally, these renoprotective effects of BM-MSCs-eRLX + GFP were maintained in the presence of perindopril co-treatment, highlighting their suitability as adjunct therapies to ACE inhibition. Importantly, no major long-term adverse effects of BM-MSCs-eRLX + GFP were observed.

**Conclusions:**

BM-MSCs-eRLX + GFP produced greater renoprotective and therapeutic efficacy over that of BM-MSCs-eGFP or ACE inhibition, and may represent a novel and safe treatment option for acute kidney injury and hypertensive CKD.

**Supplementary Information:**

The online version contains supplementary material available at 10.1186/s13287-024-03992-x.

## Background

Chronic kidney disease (CKD) is defined as kidney damage that occurs over at least 3 months and affects 1 in 10 of the general population worldwide [1]. Regardless of aetiology, kidney fibrosis is the final common manifestation of CKD and is the key contributor to kidney dysfunction and end-stage renal disease [2, 3]. Kidney fibrosis can manifest in the form(s) of tubulointerstitial fibrosis, glomerulosclerosis and/or vascular fibrosis, which disrupts normal kidney architecture and drives progressive cell death and irreversible kidney dysfunction [3, 4]. Despite this, there are currently no effective cures that can reverse or even reduce established fibrosis progression.

Human bone marrow-derived mesenchymal stem/stromal cells (BM-MSCs) have emerged as a novel treatment option for CKD owing to their immunomodulatory and tissue-reparative actions [5–7]. Substantial evidence has demonstrated the renoprotective effects of these non-immunogenic adult stem cells in various preclinical models of acute and chronic kidney injury [6], and an excellent safety profile in numerous clinical studies [8–11]. However, it has emerged that the viability of BM-MSCs post-transplantation is significantly impaired within fibrotic environments [6]. This may explain the lack of consistent efficacy of BM-MSCs in various clinical trials that have evaluated the effects of autologous or allogenic cell administration to CKD patients [8, 9]. Hence, to improve the therapeutic efficacy of BM-MSCs, various strategies have explored the genetic-engineering of cells with specific microRNAs [12], anti-inflammatory agents [13], anti-apoptotic factors [14] or angiogenic factors [15]. However, at best these strategies have had limited success in addressing the multifactorial nature of fibrosis, and do not facilitate the resolution of established fibrosis in CKD.

In light of these challenges, we have developed an innovative approach to combine BM-MSCs with recombinant human gene-2 (H2) relaxin (RLX) [6, 16], the drug-based form of H2 relaxin. The rationale for choosing RLX over other clinically-available therapies for kidney disease was based on the rapid anti-fibrotic actions it induces independently of etiology, sex or species [17–26], and over currently-used treatments for CKD [27, 28]. These effects of RLX are mediated via its suppression of transforming growth factor (TGF)-β1-induced myofibroblast differentiation and extracellular matrix (ECM) production, and promotion of matrix metalloproteinase (MMP)-induced ECM degradation.

The combined effects of RLX (0.5 mg/kg/day; via subcutaneous administration) and BM-MSCs (1 × 10^6^/mouse; via intravenous delivery ~ 15–30 min after RLX delivery) completely prevented tubulointerstitial fibrosis in mice with obstructive nephropathy [29], and more effectively attenuated interstitial fibrosis and glomerulosclerosis compared to the angiotensin-converting enzyme (ACE) inhibitor, perindopril, in a murine model of uninephrectomy followed by subcutaneous implantation of a deoxycorticosterone acetate pellet and saline to drink (1 K/DOCA/salt) for 3-weeks [28], after a 7-day treatment period. Additionally, BM-MSCs effectively lowered blood pressure (BP) to similar extent as perindopril in hypertensive mice [28]. These findings highlighted the enormous therapeutic potential of enhancing the immunomodulatory and anti-hypertensive effects of BM-MSCs with the anti-fibrotic actions of RLX in treating fibrotic kidney diseases.

However, a potential drawback to the clinical application of administering this combination therapy, is that each treatment would have to be administered separately; that is through daily injections or a microinfusion pump containing RLX [30] (due to its relatively short in vivo half-life of ~ 4–8 h [31, 32]), followed by single or multiple intravenous injections of BM-MSCs. When compared to current the standard-of-care medications that are orally consumed, this would be cumbersome and may affect patient compliance. To address this issue, we genetically-engineered and characterised BM-MSCs that expressed RLX via lentiviral transduction, to ensure constant delivery of therapeutic levels of RLX at the site of cell engraftment following transplantation. We then evaluated the anti-fibrotic and other renoprotective effects of these RLX-expressing BM-MSCs in preclinical models of acute kidney injury (which often contributes to CKD [33]) and hypertensive CKD (induced by high salt loading). In the latter model, we also compared and combined the effects of RLX-expressing BM-MSCs to that of a clinically-used frontline treatment for CKD, and evaluated the long-term safety of these engineered cells.

## Methods

### Materials

Recombinant H2 relaxin was generously provided by Corthera Inc. (San Carlos, CA, USA; a subsidiary of Novartis AG, Basel, Switzerland). Human BM-MSCs were commercially purchased from the Tulane Centre for Stem Cell Research and Regenerative Medicine (Tulane University, New Orleans, LA, USA; Website: https://medicine.tulane.edu/stem-cell-research-regenerative-medicine). Prior to any experiments being conducted on this study, these BM-MSCs were previously characterised to display a normal karyotype, form colony-forming unit-fibroblasts (CFU-F), and show multi-lineage differentiation potential [34]. These BM-MSCs were also found to express the cell surface antigens CD73, CD90 and CD105 (confirming that they were MSCs), but lacked expression of CD14, CD19, CD34, CD45 (confirming that they were not of hemopoietic or endothelial origin) and MHC class II human leukocyte antigen–DR isotype (HLA-DR; confirming that they were immunoprivileged) [34]. Perindopril Erbumine was manufactured by MedChem Express (Monmouth, NJ, USA).

### Human BM-MSC culture

Human bone marrow-derived mesenchymal stem cells (BM-MSCs) were cultured in α-minimal essential media (α-MEM) supplemented with 16% fetal bovine serum (FBS), 1% L-glutamine (2 mM) and 1% penicillin/streptomycin (100U/ml) (all from Thermo Fisher Scientific, Scoresby, Victoria, Australia) at 37 °C, and were seeded at a density of 60 cells/cm^2^ in tissue culture flasks (BD Falcon, North Ryde, New South Wales, Australia) with media replaced every 3–4-days until 70–80% confluency was reached. Passages 3–4 (P3-P4) cells were used for transduction.

### Lentiviral production

**Fig. 1:**
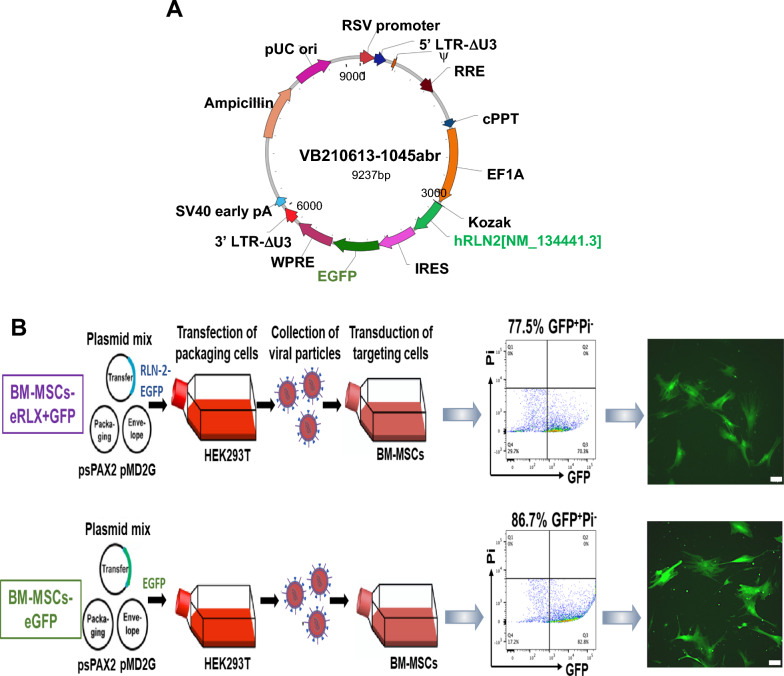
Design, transduction and characterisation of BM-MSCs-eRLX + GFP. **A** Shows a schematic illustration of the pLV-EF1A-hRLN2-EGFP vector that was designed and transduced into BM-MSCs. The vector comprised bicistronically expressed human relaxin-2 cDNA and EGFP genes linked by internal ribosome entry site (IRES), under an EF1A promoter. **B** Lentivirus was produced with a three-plasmid expression system with transfer (which contained the *RLN-2-EGFP* genes or *EGFP* gene of interest), packaging and envelope plasmids in HEK293T cell lines, before being transduced into passage (P)3–4 BM-MSCs at a MOI of 2. After flow sorting, the successfully transduced cells (GFP^+^) were expanded and passaged until P6, when they were subjected to flow cytometry analysis of GFP^+^ cells to test the long-term stability of lentiviral transduction, which represented averages of ~ 77.5% and ~ 86.7% of BM-MSCs transduced with virus containing pLV-EF1A-hRLN2-EGFP or pLV-EF1A-EGFP, respectively. Representative fluorescent microscopy images also confirmed the GFP expression in the P6 BM-MSCs-eRLX + GFP and BM-MSCs-eGFP, respectively. Scale bar = 100 µm

To expand pLV-EF1A (eukaryotic translation elongation factor-1α)-hRLN2-EGFP lentiviral vector DNA (Fig. 1A), ~ 1 mL (75 ng) of vector DNA was mixed with ~ 10uL of one-shot stb13 competent E.coli (C737303; Thermo Fisher Scientific, Scoresby, Victoria, Australia), and incubated for 30 min, before subjected to heat shock in a water bath for 30 s at 42 °C, and incubated on ice immediately following that. Bacteria were then transferred to 500µL SOC medium (15,544,034; Thermo Fisher Scientific) and shaken at 37 °C for 1 h at 225 rpm, before 250µL was spread onto a pre-warmed ampicillin (50µL/mL) LB-amp plate and incubated overnight at 37 °C. On the following day, one colony was picked into 5-7 mL LB-Amp broth to expand in a shaker (for 8 h at 225 rpm, at 37 °C), before being diluted at 1:1000 with 100 mL LB-Amp broth and incubated in 37 °C in a shaker at 250 rpm for 16 h. The next day, plasmid DNA was extracted and purified using a QIAGEN Plasmid Plus Midi Kit (#12,943 and #12,945) following the manufacturer’s protocol. The DNA concentration of the pLV-EF1A-hRLN2-EGFP and pLV-EF1A-EGFP plasmids were 1483 ng/µL and 553 ng/µL, respectively.

### Lentiviral expansion and titre determination

Lentiviral particles containing pLV-EF1A-hRLN2-EGFP or pLV-EF1A-EGFP were produced in HEK293T cells by transient co-transfection using the three-plasmid expression system. Briefly, 17.5 million cells were plated into a T175 flask a day before transfection and 24 h later, the culture medium was replaced by supplemented advanced DMEM containing 2% FBS (Invitrogen, Mount Waverley, Victoria, Australia), 1% non-essential amino acids (NEAA; Sigma-Aldrich, Castle Hill, New South Wales, Australia), 1% GlutaMax (2 mM; Invitrogen, Australia) and 1% penicillin/streptomycin (100U/ml) prior to the addition of transfection mix containing transfer vector DNA (with human relaxin-2 gene), packaging plasmid DNA (psPAX2; Addgene Plasmid #12,260) and envelope plasmid DNA (pMD2.G; Addgene plasmid #12,259) at a 2.5:1.6:1 ratio in 1.25 ml of sterile H_2_0 per T175 flask, which contained 4.5 µg PEI/µg total DNA. The medium was replaced after 16–24 h (on day-2) and again after 48 h (on day-3) post-transfection. On day-3, the conditioned medium containing the lentiviral particles was collected into sterile 50 mL tubes. To increase the viral titer, the cell culture supernatant containing lentiviral particles, at 72 h post-transfection, was filtered through a 0.45 µm stericup-HV sterile vacuum filtration system (SCHVU01RE; Millipore Merck, Darmstadt, Germany) and transferred to Millipore concentrators with a 100 kDa cut-off (Amicon®Ultra-15, Merck Millipore, Tullagreen, Cork, Ireland) and centrifuged repeatedly for 20–30 min at 4000 rpm at 4 °C until the sample of each supernatant was concentrated to ~ 200μL. The concentrated samples containing lentiviral particles were finally transferred to 0.2 ml Eppendorf tubes and stored at -80 oC until required.

To determine the virus titre (the number of host cells per unit virus can infect), cryopreserved BM-MSCs were seeded in 12-well plates at a density of 5 × 10^4^ cells per well. After incubation at 37 °C for 24 h, the culture medium was replaced with 0.5 mL of virus particles diluted by 1000-, 10,000- and 100,000-fold in α-MEM supplemented with 2% FBS, 1% L-glutamine (2 mM) and 1% penicillin/streptomycin (100U/ml), in the presence of polybrene transfection reagent (1:1700; TR-1003-G; EMD Millipore, Merck KGaA, Darmstadt, Germany) in duplicate. Notably, cells that were transduced with pLV-EF1A-hRLN2-EGFP and pLV-EF1A-EGFP were termed BM-MSCs-eRLX + GFP and BM-MSCs-eGFP (as a control engineered cell type), respectively. The culture medium was replaced with 1 mL of MSC medium every 24 h. At 72 h post-transduction, transduction efficiency was measured by detecting % GFP-positive (GFP^+^) cells on a LSR Fortessa flow cytometer at a concentration of 2.5 × 10^5^ cells/mL in FACS buffer (0.5% BSA, 0.5% 0.5 M EDTA in PBS) with propidium iodidie (PI; 1:500; P4864; Sigma-Aldrich). The transduction efficiency (F) was then used to calculate the virus titer (infectious units per µl of virus), by multiplying the number of targets MSCs (Cn), and dividing that by the volume of input virus (V).

### BM-MSC transduction

Cryopreserved human BM-MSCs were plated at a density of 5 × 10^4^ cells per well in a 12-well plate. According to the virus titre calculated using the formula described above (1.58 × 10^7^TU/ml and 1.5 × 10^8^TU/ml for Lentiviral particles containing pLV-EF1A-hRLN2-EGFP or pLV-EF1A-EGFP, respectively), transduction was performed with a multiplicity of infection (MOI; the ratio of infectious virions to cells) of 2, for both cell lines, at a final volume of 0.5 ml per well using the same method described above. Cells were sorted on the BD influx system flow cytometer at 72 h post-transduction. The GFP^+^ cells from the cultured BM-MSCs-eRLX + GFP and BM-MSCs-eGFP, respectively were collected and cultured until 80% confluency was reached (after ~ 7–10-days), before they were used for assessment of cell proliferation, cell differentiation, measurement of RLX levels using the human relaxin-2 ELISA or passaged for expansion. For long-term storage, cells were trypsinized, pelleted and resuspended in a freezing medium containing 30% FBS, 1% penicillin/streptomycin, and 5% dimethylsulfoxide (DMSO; Sigma-Aldrich, St Louis, MO, USA), frozen at -1 °C/min until reaching -80 °C, before being stored in liquid nitrogen. To ensure the stable transduction of both lentiviral vectors in long-term culture, flow cytometry was performed on a LSR Fortessa flow cytometer after 2 passages of flow sorting (P6) to quantify % GFP^+^ cells within BM-MSCs-eRLX + GFP and BM-MSCs-eGFP populations again, which represented ~ 77.5% and ~ 86.7% of each cell population, respectively (Fig. 1B), confirming the long-term incorporation of the vector construct in BM-MSCs using the transduction method used.

### RXFP1 staining of BM-MSCs-eRLX + GFP

RXFP1 expression on BM-MSCs-eGFP (as control transduced cells) and BM-MSCs-eRLX + GFP was determined by immunofluorescence staining. Briefly, 4 × 10^4 ^cells, respectively, were cultured on a 12 mm glass coverslip (pre-coated with 0.1% gelatin) in a 24-well plate. Following overnight incubation at 37 °C, cells were fixed in 4% PFA for 10 min at room temperature, washed in PBS for 5 min (3 times) and blocked for non-specific protein binding in 2% Bovine serum albumin (BSA) for 1 h at room temperature. The fixed cells were then washed again in PBS for 5 min (3 times) and incubated with a rabbit polyclonal RXFP1 antibody (A9227 [35]; 1:2000 dilution; Immunodiagnostik AG, Bensheim, Germany) in 2% BSA overnight at 4 °C, whilst the negative control was incubated only with 2% BSA. On the second day, the primary antibody was washed with PBS, before cells were incubated with goat anti-rabbit Alexa-fluor 555 secondary antibody (A21428; 1:500; Invitrogen, Scoresby, Victoria, Australia) in 2% BSA for 1 h at room temperature, before washed 3 times with PBS. Nuclear counterstaining was performed by adding ~ 10ul of VECTASHEILD@ Antifade Mounting Medium (With DAPI; H-1200, Vector Laboratories) prior to coverslipping. Immunoreactive RXFP1 (red staining) was visualised at × 200 magnification using the Leica AF6000LX microscope, in association with the GFP^+^ cells (green) and DAPI (blue)-stained cells, and merged using ImageJ software.

### Proliferation assay

To assess whether viral transduction of BM-MSCs affected cell viability and proliferative capacity, BM-MSCs were seeded into 96-well plates at 5 × 10^3^ cells per well containing 100 µl of culture media, or media supplemented with RLX at 1 ng/ml or 10 ng/ml (in triplicate), and cultured at 37 °C, for 3- or 7-days. Separate sets of triplicate wells were used to culture BM-MSCs-eRLX + GFP or BM-MSCs-eGFP under the same condition. After 3- and 7- days of culture, cell proliferation was determined using the CellTiter 96 AQ_ueous_ One Solution Proliferation Assay (Promega, Fitchburg, WI, USA) according to the manufacturer’s instructions.

### Differentiation assay

To assess whether RLX-expressing BM-MSCs had the multipotency of naive BM-MSCs, the adipogenic, osteogenic and chondrogenic potential of BM-MSCs-eRLX + GFP were assessed using the Human Mesenchymal Stem Cell Functional Identification Kit (SC006; R&D Systems, Minneapolis, MN, USA) at passage 6 (P6), as per the manufacturer's instructions. Adipocytes, osteocytes and chondrocytes were identified via positive histological staining with Oil-red-O, Alizarin red and alcian blue, respectively; or via immunohistochemical staining for fatty acid binding protein 4 (FABP4), osteocalcin and aggrecan, respectively. Adipocytes and osteocytes that were subjected to histological and immunofluorescent staining were imaged at × 200 magnification using an Olympus fluorescent microscope and CellSens Software (version 1.15; Olympus, Bartlett, TN, USA), whereas the chondrocytes pellets were imaged with a fluorescent microscope (Provis AX70; Olympus).

### Migration assay

The migration capacity of naïve BM-MSCs, in the absence of presence of RLX (10 ng/ml), as well as BM-MSCs-eRLX + GFP or BM-MSCs-eGFP was determined over a 24 h (hr) period. To perform the assay, 2 × 10^4^ cells were suspended in 70µL growth media and seeded into 2 well-silicone in 35 mm dishes for wound healing assay (Ibidi Inc., Fitchburg, WI, USA) and incubated at 37 °C for 24 h to form a confluent cell layer. Artificial wounds within the dishes were then created at a gap distance of 500 ± 100 µm [35]. The dishes were replenished with 2 mL of MSC growth media supplemented with various treatments of interest. The number of cells that migrated to close the wound was assessed by using light microscopy. Photomicrographs of the migrated cells were captured at 0-, 2-, 4-, 6-, 12- and 24-h post-treatment. The percentage of gap closure area was determined using ImageJ software (NIH, Bethesda, MD, USA). The slope of the linear phase was used to characterise the average wound closure rate and was expressed as a % of that measured from the migration capacity of naïve BM-MSC controls.

### Secretion profile of BM-MSCs-eRLX + GFP compared to naïve BM-MSCs

**Fig. 2:**
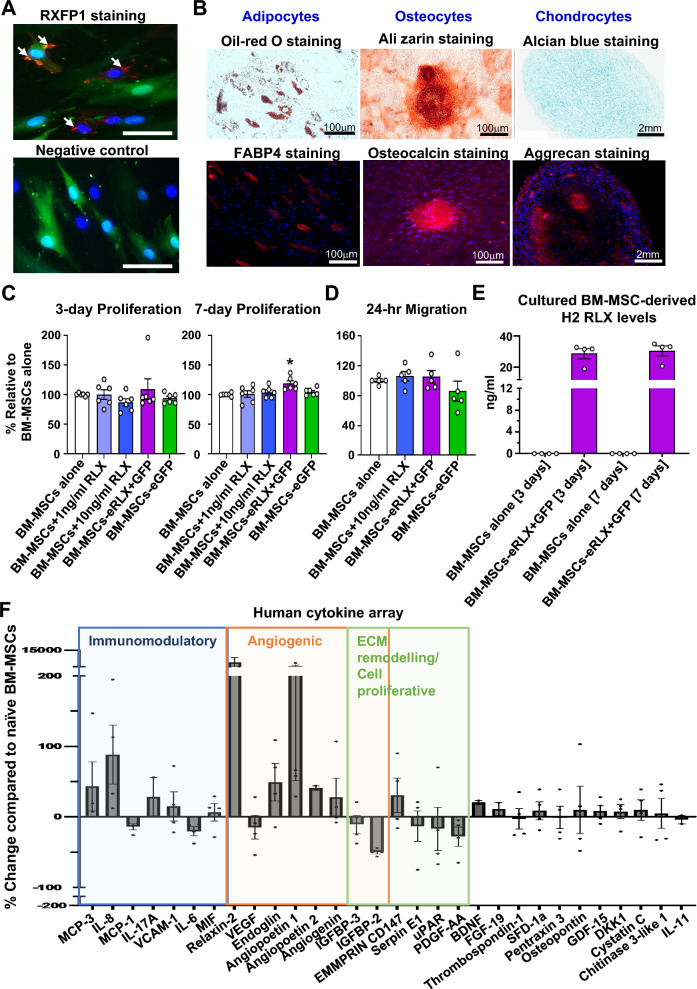
Proliferation, migration and cytokine-secretion capacity of BM-MSCs-eRLX + GFP. **A** RXFP1 (red staining) was detected on the cell surface of BM-MSCs-eRLX + GFP; which was colocalised with DAPI-nuclear (blue) staining. A negative control image is also provided in which the cells shown were not stained with the primary RXFP1 antibody used. **B** Representative images show the trilineage differentiation ability of BM-MSCs-eRLX + GFP (as per their naïve/unmodified counterparts) to differentiate in vitro into adipocytes, osteocytes and chondrocytes, as confirmed with histological and immunohistochemical staining (for adipocytes using oil-red O and fatty acid binding protein 4 (FABP4) staining; osteocytes using alizarin red and osteocalcin staining; and chondrocytes using alcian blue and aggrecan staining) colocalized with DAPI-staining. Scale bar = 2 mm or 100 µm as indicated. **C **The mean ± SEM 3-day and 7-day proliferation capacity of BM-MSCs alone or treated with 1 ng/ml or 10 ng/ml RLX vs BM-MSCs-eRLX + GFP or BM-MSCs-eGFP, and **D** 24-h migration capacity of these cells (expressed relative to that of the untreated naïve BM-MSCs). *p < 0.05 vs the (unstimulated) BM-MSCs group; as determined using a one-way ANOVA and Tukey’s *post-hoc* test. **E** The ELISA-determined levels of H2 RLX that were produced by naïve BM-MSCs (n = 4) vs BM-MSCs-eRLX + GFP (n = 4) that were cultured for 3- and 7-days in vitro are also shown. **F** Additionally, 30 cytokines that were measurably altered (up- or down-regulated; as determined by a human cytokine array with antibodies to 105 cytokines) in the cell supernatant of BM-MSCs-eRLX + GFP that were cultured for a 7-day period are shown, which were expressed as the % change relative to their naïve BM-MSCs counterparts. Data are presented from n = 4 separate experiments, and cytokines that play a role in immunomodulation, angiogenesis (tissue repair) and ECM remodeling/cell proliferation are grouped and highlighted. Data points between 200 and 15,000 are not shown due to the break in the y-axis. Abbreviations: MCP, monocyte chemoattract protein; IL, interleukin; VCAM-1, vascular adhesion molecule-1; MIF, macrophage migration inhibitory factor; VEGF, vascular endothelial growth factor; IGFBP, insulin growth factor binding protein; uPAR, urokinase-type plasminogen activator receptor; PDGF-AA, platelet-derived growth factor containing two A subunits; BDNF, brain-derived neurotrophic factor; FGF, fibroblast growth factor; SDF, stromal cell-derived factor; GDF, growth differentiation factor; DKK1, Dickkop Wnt signaling pathway inhibitor 1

To characterise the secretion profile BM-MSCs-eRLX + GFP, a human cytokine array was performed using a Proteome Profiler Human XL Cytokine Array Kit (ARY022B; R&D Systems), from the 7-day cell culture supernatant of BM-MSCs-eRLX + GFP and naïve BM-MSCs (from n = 4 separate supernatant samples per cell type). Briefly, the nitrocellulose membranes (containing capture and control antibodies spotted in duplicate) were blocked for 1 h, before incubating with 400µL of cell culture supernates (from 0.5 × 10^6^ cells of each type) overnight at 4 °C on a rocking platform shaker. The membrane was then incubated for 1 h with the detection antibody cocktail, washed and incubated with streptavidin-HRP. Finally, the Chemi Reagent mix was spread on the membrane to obtain an autoradiograph, and the duplicate dots were quantified by densitometry using a ChemiDoc MP imaging System (Bio-Rad; South Granville, New South Wales, Australia). The OD of each duplicate dot (representing one cytokine/chemokine; see mean ± SEM OD of the cytokine/chemokine intensity detected in STable 1 [Supplementary File]) from BM-MSCs-eRLX + GFP was corrected for that of the reference levels for each membrane and expressed relative to the corresponding values from naïve BM-MSCs (% change compared to that secreted by naïve BM-MSCs alone), which was expressed as 0 in each case. Only the % change from the detectable cytokines are presented in Fig. 2F.

### Exosome extraction from BM-MSCs-eRLX + GFP

P6 BM-MSCs-eRLX + GFP were grown to approximately 80% confluency and starved in DMEM supplemented with 1% FBS and 1% penicillin/streptomycin for 72 h. Media was collected and clarified at 2000 g for 30 min, followed by 0.45 μm filtration. Clarified media was ultracentrifuged (Sorvall WX 100 + Ultracentrifuge; Beckman Coulter Type 70.1 Ti fixed-angle rotor) at 100,000 g for 1 h at 4 °C (Beckman Coulter Australia, Mount Waverley, Victoria, Australia). The top 9 ml of each tube was discarded, the bottom 1 mL was pooled and spun again using the same parameters, and the rest (conditioned medium) was stored at -80 °C. Media was aspirated, and the sides of the tubes were washed thoroughly with 500μL of PBS. Particle size and yields were determined using the Zetaview® PMX-120 Nanoparticle Tracking Analyzer (NTA) (Laser = 488 nm, Sensitivity = 80 AU, Shutter Speed = 100 s^−1^, Frame Rate = 30/s). Analysis was conducted on a 1:100 dilution of isolated exosome stock (Max Area = 1000px, Min Area = 10px, Max Brightness = 30). A flowchart depicting the process of exosome extraction and detailing the size of the exosomes extracted is shown in Figure S1 [Supplementary File]. The extracted exosomes were lysed with RIPA Buffer (#9806; 10x) containing proteinase inhibitor (#5872; 100x) and PMSF (#8553; 200x; all from Cell Signaling Technology; Danvers, MA, USA) for measurement of H2 RLX levels.

### Animals

All animal care and experimental procedures performed adhered to the NIH guidelines for the Care and Use of Laboratory Animals for Scientific Purposes, and were approved by the Animal Ethics Committee of Monash University. The work has been reported in line with the ARRIVE guidelines 2.0. The sex of animals was considered in the study design: given that male mice are more prone to undergo a more rapid rate of kidney disease progression and hypertensive renal injury, in line with hypertension and CKD being more common in men compared to women [36], male mice were used since they provided a larger therapeutic window in which the efficacy of cell-based treatments evaluated could be measured. Initially, groups of equal size were designed using randomization and blinded analysis; although confounding variables were not controlled. 6-week-old male C57BL/6 J mice were obtained from Monash Animal Services (Monash University, Clayton, Victoria, Australia) to compare the renoprotective effects and biodistribution of BM-MSCs-eGFP versus BM-MSCs-eRLX + GFP in a model ischemia reperfusion injury (IRI; under animal ethics number: MARP/2022/29417). Mice subjected to IRI were anaesthetised with inhaled isoflurane (2–3% in oxygen; Baxter Healthcare) and provided analgesia (carprofen; s.c) just prior to surgery, and 24 h and 48 h post-surgery. 11–12-week-old male Balb/c mice were obtained from Monash Animal Services (under animal ethics number: MARP/2021/30019) to compare the therapeutic effects, biodistribution and safety of BM-MSCs-eGFP versus BM-MSCs-eRLX + GFP in a model of high salt (HS)-induced hypertensive nephropathy; which did not require any anaesthesia or analgesia. In the latter model, the therapeutic effects of BM-MSCs-eRLX + GFP were also compared to, or combined with, the current standard of care medication, perindopril. All mice were housed (in groups of four per cage) under a controlled environment, given a 5- to 6-day acclimatisation period, and maintained on a 12-h light/12-h dark cycle with free access to normal rodent lab chow (Barastock Stockfeeds, Pakenham, Victoria, Australia) and water.

In each case, power calculations were performed to ensure that adequate group sizes were used for the studies conducted; where it was determined that with a 20–25% standard deviation, we would be 80% powered to detect a 23–28% effect with n = 8 animals per group. In either (the IRI or HS) model established, the effects of RLX alone was not assessed, since our previous work had already determined that the combined effects of RLX and BM-MSCs provide broader renoprotection over that of RLX alone in normotensive [29] and hypertensive [28] models of kidney disease.

### Induction and treatment of a murine model of ischemia–reperfusion injury (IRI)

To assess the therapeutic effects of BM-MSCs-eRLX + GFP as a treatment for AKI, a unilateral IRI-induced model of AKI was established in 6-week-old male C57BL/6 J mice (n = 32), weighing approximately 25-30 g. Mice were initially anaesthetised with 4% isoflurane in an induction chamber, and then maintained with 1–2% isoflurane via a nasal cone for the remainder of the surgery. Using a vascular clamp (0.4–1.0 mm; Fine Science Tools, Heidelberg, Germany), the renal pedicle (which included the renal artery and vein) was identified and clamped for 40 min. After 40 min, the clamp was removed and during this reperfusion stage, subgroups of mice were either i) left untreated (IRI group; n = 8) or given an intra-renal injection of ii) 1 × 10^6^ BM-MSCs-eGFP (n = 8) or iii) 1 × 10^6^ BM-MSCs-eRLX + GFP cells (n = 8) (all suspended in 200 µl PBS). iv) To compare the renoprotective effects of BM-MSCs-eRLX + GFP cells to that of BM-MSCs and RLX administered separately, a separate group of mice (n = 8) had a small dorsal incision made during the ischemic period, in order to implant 7-day osmotic mini pumps (Model 1007D; with a release rate of 0.5 µλ/hour; ALZET, Cupertino, California, USA) containing RLX (Pump-RLX; 0.5 mg/kg/day). This dose of RLX had previously been found to produce circulating RLX levels of ~ 18-20 ng/ml in mice after 5-days [37]. During the reperfusion stage, these mice were injected with 1 × 10^6^ BM-MSCs (suspended in 200 µl PBS). v) A sham-operated control group (n = 8) was also included with mice being anaesthetised and left flank incision made to expose the kidney, but the kidney remained untouched. The four groups of mice were maintained until 7-days post-sham or IRI surgery, before being killed for blood and kidney tissue collection and analysis.

Additionally, the in vivo tracking of transplanted BM-MSCs-eGFP and BM-MSCs-eRLX + GFP was conducted on a separate cohort of IRI mice (n = 24), which were either subjected to IRI alone (n = 2 per time point) or were subjected to IRI and received a single i.v-injection of BM-MSCs-eGFP (n = 3 per time point) or BM-MSCs-eRLX + GFP (n = 3 per time point). As our previous work had determined that i.v-administered BM-MSCs-eGFP immediately migrated to the lungs of IRI mice, but then homed to the injured kidney after 24 h and remained in the kidney after 72 h [34], IRI mice treated with BM-MSCs-eGFP or BM-MSCs-eRLX + GFP were then killed at 5-, 7- and 14-days post-transplantation for the analysis of the biodistribution of cells, and quantification of GFP^+^ cells within the kidney. At the appropriate time-points, mice were killed with an overdose of inhaled isoflurane (5% in oxygen) followed by cardiac puncture and exsanguination, so that blood and appropriate organs could be isolated for analysis.

### Induction and treatment of a murine model of high salt (HS)-induced hypertensive kidney disease

To assess the therapeutic effects of BM-MSCs-eRLX + GFP as a treatment for hypertensive CKD, a HS (2% NaCl in drinking water)-induced model of hypertension was established in 11–12-week-old male Balb/C mice (n = 40; weighing 20-25 g) for 8-weeks; which was adapted from a previously published study [38]. Balb/c mice exhibited hypertension, kidney inflammation and fibrosis after 6-weeks of 2% NaCl drinking [38]. We therefore employed an 8-week model of HS loading, from which sub-groups of HS-fed mice were treated during the final two-weeks to assess the therapeutic impact of various treatments in mitigating the established salt-sensitive hypertension and related renal pathologies. The i) control group (n = 8) was maintained on normal drinking water (NDW) for the 8-week period. At week-7 post-HS loading, subgroups of the n = 48 HS-fed mice were randomised to either receive ii) no treatment (HS group; n = 8); or an intravenous (i.v; tail vein) injection of iii) 1 × 10^6^ BM-MSCs-eGFP (and then again on week-8 (× 2); n = 8); iv) BM-MSCs-eRLX + GFP (× 1; n = 8); v) BM-MSC-eRLX + GFP (× 2; then again on week-8; n = 8); vi) Pump-RLX delivered via an s.c. implanted 14-day osmotic minipump (0.5 mg/kg/day; Model 2002; with a release rate of 0.5 µl/hour; ALZET) and i.v. injection of BM-MSCs (1 × 10^6^/mouse; and then again on week-8) (Pump-RLX + BM-MSCs; n = 8); or vii) the angiotensin-converting enzyme inhibitor, perindopril (2 mg/kg/day via drinking water; a dose that induced anti-hypertensive effects without inducing weight loss [28, 39]; n = 8) via drinking water.

In a separate study that assessed the renoprotective effects of BM-MSCs-eRLX + GFP (× 2) in the presence of perindopril treatment, control groups of mice that were maintained on NDW (n = 8) or HS (n = 24; n = 8 subjected to HS alone) for 8-weeks were again established. Additional sub-groups of HS-fed mice were then subjected to perindopril alone (as above; n = 8); or perindopril + BM-MSCs-eRLX + GFP (× 2; n = 8). In both studies, all treatments were maintained for two-weeks, from weeks-7–8 post-HS, before all groups of mice were killed by an overdose of inhaled isoflurane (5% in oxygen) followed by cardiac puncture and exsanguination, for blood, heart and kidney tissue collection and analysis.

### Safety study

To assess the long-term safety of BM-MSC-eRLX + GFP (× 2) treatment, a further set of HS-fed (n = 9) mice that were either untreated (n = 3) or treated with BM-MSC-eRLX + GFP (× 2; n = 6) were established and treated as outlined above; whilst NDW-fed mice (n = 3) were also included as controls. All mice were maintained on NDW or HS drinking for 8-weeks (as above), but were then maintained on NDW for the further 9-months (39-weeks) after treatment cessation (to eliminate the long-term effects of HS-drinking as a confounding variable). At the completion of this period, when mice were 59-weeks of age, mice were transported to an independent Pathology company (Cerberus Sciences, Scoresby, Victoria, Australia) for histopathological assessment of various organs. The kidneys, adrenal glands, heart, aorta, lungs, thyroid gland, liver, spleen, stomach, pancreas, mesenteric lymph node, gall bladder, brain and skin of all mice were histologically assessed for abnormalities. Interstitial kidney fibrosis was also measured from the four groups of mice that survived the 9-month treatment cessation period; by Masson’s trichrome staining of interstitial ECM deposition.

### SBP measurements

Systolic blood pressure (SBP) measurements of all NDW and HS-fed mice were measured via tail cuff plethysmography, using the multichannel MC4000 Blood Pressure Analysis System (Hatteras Instrument Inc; Cary, NC, USA), at baseline and then fortnightly over the 8-week experimental period; and monthly over the 9-month treatment cessation period (for the safety study). At least 20 measurements per time point were pooled to obtain a mean value for each animal.

### Confirmation of BM-MSC homing to the kidney

To determine if the i.v-injected engineered cells could be detected in the kidneys and other organs of IRI or HS-fed mice, the left kidney pole, heart, lungs, spleen and liver of IRI mice; and left kidney pole and heart of HS-fed mice that were treated with BM-MSC-eGFP or BM-MSC-eRLX + GFP were subjected to genomic DNA (gDNA) extraction using a DNeasy Blood&Tissue kit (Qiagen, Clayton, Victoria, Australia) following the manufacturer's protocol. The extracted gDNA was then used for the analysis of *EGFP* gene expression by polymerase chain reaction (PCR). gDNA from 1 × 10^6^ BM-MSCs-eGFP that were not injected into mice was used as a positive control. On the other hand, the replacement of gDNA with distilled water was included as a negative control. The PCR mixture contained the following components, including 10µL GoTaq Green Master Mix (Ref: M712B; Promega; Madison, WI, USA), 1 µL forward primer: 5’-TAC GGC AAG CTG ACC CTG AAG TTC-3’and 1 µL reverse primer: 5’CGT CGT CCT TGA AGA AGA TGG TGC G-3’ primer pairs (Integrated DNA Technologies, Singapore), 1 µL DNA template and 7 µL of Nuclease-free water. PCR mixtures were run under the following conditions: 94 °C for 2 min, 35 cycles (of denaturing for 30 s at 94 °C, annealing for 30 s at 60 °C, and extending for 1 min at 72 °C), then 72 °C for 7 min, before samples were held at 10 °C before collection. PCR products were then separated by electrophoresis on a 2% (w/v) agarose gel, where the primer sequences used were expected to produce a 196 base-pair product. Notably, 100 bp or 50 bp DNA ladders were used for gels containing DNA samples extracted from IRI or HS-fed mice, respectively. Gels were imaged with a ChemiDoc MP imaging System (Bio-Rad; South Granville, New South Wales, Australia).

## Histology and immunohistochemistry

Serial paraffin-embedded left kidney Sects. (4 µm thickness; from the kidney mid-zone of IRI and HS-fed mice) were processed and stained with either haematoxylin and eosin (H&E) for the semi-quantification of inflammatory cell infiltration and proximal tubular damage [40]; picrosirius red-staining of interstitial collagen deposition [29, 41]; or Masson’s trichrome staining of interstitial ECM deposition (for the safety study only) or glomerular matrix deposition (glomerulosclerosis) [28, 41], respectively. To visualize the localization of GFP^+^ cells in the kidneys from animals treated once-weekly with BM-MSC-eGFP (× 2) or BM-MSC-eRLX + GFP (× 2), separate left kidney frozen sections (from one kidney pole of these two groups) embedded in optimal cutting temperature (OCT) were sectioned into 6 µm thickness, fixed in 4% paraformaldehyde (PFA), and mounted with VECTASHEILD® Antifade Mounting Medium (with DAPI; H-1200, Vector Laboratories). Separate 6 µm frozen sections from the kidneys of NDW-fed mice (negative control) were also visualized. GFP^+^ cells within each section were imaged at × 400 magnification, using the DAPI filter for visualization of cell nuclei (blue staining), with a Leica AF6000LX microscope. To quantify the homing of the engineered cells, the number of GFP^+^ cells/field of view was determined in the kidneys of mice treated with once-weekly injections of BM-MSC-eGFP (× 2) or BM-MSC-eRLX + GFP (× 2), from 6 non-overlapping fields at × 200 magnification. Notably, the kidney samples were also stained with a goat anti-collagen IV polyclonal IgG (#1340–01;100 dilution; SouthernBiotech, Birmingham, AL, USA) followed by a donkey anti-goat Alexa Fluor™ 555 IgG (A21432; 1:500 dilution; Invitrogen) secondary antibody, to outline the basement membrane (red-staining) so that the location of GFP-labelled cells within the injured kidneys of mice could be determined.

Further serial paraffin sections obtained from the kidneys of IRI and HS-fed mice were also immunohistochemically (IHC) stained with either a rat monoclonal IgG2b antibody, Clone 222,414 (#MAB1817, 1:100 dilution; R&D Systems) to kidney injury molecule-1 (KIM-1; a marker of tubular epithelial injury), a rabbit polyclonal antibody (#ab5694, 1:1000 dilution; Abcam Antibodies, Cambridge, MA, USA) to α-smooth muscle actin (α-SMA; a marker of myofibroblast differentiation) or a rabbit polyclonal antibody (#ab28364, 1:150 dilution; Abcam Antibodies) to CD31 (an endothelial cell surface marker that was used to evaluate peritubular capillary density), followed by incubation with an anti-rat (#7077; 1:500 dilution; Cell Signaling Technology, Danvers, MA, USA) or anti-rabbit (#K4003; Agilent Technologies) secondary antibody, respectively. Additionally, the localization of pSmad2 in the kidney of HS-fed mice was semi-quantitated with immunofluorescent staining, using a rabbit monoclonal antibody to detect pSmad2 expression (#3108S), followed by exposure of sections to a goat polyclonal anti-rabbit Alexa Fluor™ 594 IgG secondary antibody (1:500; Invitrogen). Furthermore, a rat monoclonal IgG2b antibody, clone CI:A3-1 was IF-stained to detect F4/80^+^ macrophage accumulation (#MCA497R, 1:100 dilution; Bio-Rad Laboratories, Portland, ME, USA), which was co-stained with a goat anti-collagen IV polyclonal IgG to visualize co-localization of F4/80^+^ macrophages with type IV collagen (to outline the kidney structure; #1340–01; 1:100 dilution; SouthernBiotech, Birmingham, AL, USA), followed by staining with goat anti-rat Alexa Fluor™ 488 IgG (A11006;1:500 dilution; Invitrogen) and donkey anti-goat Alexa Fluor™ 555 IgG (A21432; 1:500 dilution; Invitrogen) secondary antibodies, respectively.

All slides subjected to histological, IHC or IF staining detailed above were scanned and captured with the Aperio Scanscope AT Turbo (Leica Microsystems Pty, Ltd, VIC, Australia) and assessed with Image J (FIJI) software (NIH, Bethesda, MD, USA). Morphometric analysis of each kidney section was randomly performed on 8–10 non-overlapping fields of view per section, at × 400 magnification by a blinded investigator. Total kidney inflammatory cell infiltration was semi-quantitated from H&E-stained kidney sections on a scale of 0–4 as previously described [40]. Tubulointerstitial fibrosis was determined from picrosirius red-stained kidney sections and was expressed as the % stained area per field analysed [29, 41]. To further assess glomerulosclerosis using Masson’s trichrome-stained kidney sections, ~ 30 glomeruli were randomly selected from each section, with the severity of glomerulosclerosis scored based on a previously described method (from a scale of 0–4) [42]. All IHC- or IF-stained slides were semi-quantitated by the percentage of positive DAB (brown) staining or positive fluorescent (green) staining relative to the total area of the field analysed, respectively.

### Flow cytometry

Flow cytometry was performed on cell suspensions from freshly isolated whole kidneys of NDW controls (n = 6), HS-fed mice (n = 6) and HS-fed mice treated once-weekly with BM-MSC-eRLX + GFP (× 2; n = 6). Briefly, the right kidney of each mouse was digested mechanically using fine scissors, and then enzymatically by incubation for 30 min with 2 mL of kidney dissociation medium containing collagenase/dispase (3 mg/ml; 10,269,638,001; Roche), DNAse type 1 (0.02%; 11,284,932,001; Roche) and CaCI2 (0.05 mM) in sterile HANKS balanced salt solution. The digested tissue was passed through a 70 mm sterile filter (BD Biosciences, USA), before 10 ml of flow cytometry buffer (FACS buffer; PBS supplemented with 0.5% BSA and 5 mM EDTA) was added to stop the enzymatic reaction. Cells were pelleted by centrifugation at 400 g (at 4 °C) for 5 min and re-suspended in 2 ml RBC lysis buffer for 1 min at room temperature, before topping up with cold FACS buffer to 10 ml and centrifuging again for 5 min at 400 g. Finally, the cell pellet was resuspended in 2 ml of FACS buffer, in which 10 ul was subjected to cell counting. Samples were then centrifuged at 400 g for 4 min and stained with the fluorochrome-conjugated antibody cocktail (listed in STable 2 [Supplementary File]) for 20 min at 4 °C in the dark. FcR blocking reagent (1:100 dilution, Miltenyi Biotec, Bergisch Gladbach, Germany) was used to exclude any non-specific binding of antibodies to cells expressing the Fc receptor. The stained cells were centrifuged at 400 g for 4 min, and washed and resuspended in FACS buffer with PI (1:500). All samples were analysed using Fortessa X20 instrument controlled by FlowDiva software (BD Biosciences, Melbourne, Victoria, Australia). Data were analysed using FlowJo software (see Figure S2 [Supplementary File] for the gating strategy used).

### Enzyme-linked immunosorbent assay (ELISA)

The H2 RLX-specific Quantikine ELISA (DRL200; R&D systems, Minneapolis, MN, USA) was used to assess RLX levels in the supernatants of 1 × 10^6^ BM-MSCs-eRLX + GFP cultured for 3- or 7-days; from the conditioned medium and 1–1.25 × 10^9^ lysed exosomes collected from BM-MSCs-eRLX + GFP; and from the circulation of IRI- or HS-fed that were treated with BM-MSCs-eGFP or BM-MSCs-eRLX + GFP.

Total protein was also extracted from an injured kidney pole of IRI and HS-fed mice, using a previously described method [43]. Mouse Duoset ELISA kits (R&D Systems) were used to assess the expression of the pro-inflammatory markers: monocyte chemoattractant protein (MCP)-1 (DY479-05), tumour necrosis factor (TNF)-α (DY410-05) and interleukin (IL)-1β (DY401-05) from HS-fed mice; and the pro-fibrotic marker: transforming growth factor (TGF)-β1 (DY1679-05) in both in vivo models established. In all cases, duplicate aliquots per sample were assessed by ELISA analysis in accordance with the manufacturer’s instructions. All endpoints were expressed as ng/ml.

### Gelatin zymography

The same tissue extracts (used for ELISA analysis) from HS-hypertensive mice were separately used to assess the activity of MMP-2 (gelatinase A) and MMP-9 (gelatinase B) by gelatin zymography. Briefly, equal amounts of kidney extracts containing 10 µg of protein per sample were loaded on 7.5% acrylamide gels containing 1 mg/ml gelatin as described previously [28, 43]. Gelatinolytic activity of glycosylated (G), latent (L) and active (A) MMP-2 or -9 was each visualized as clear bands and the optical density (OD; in arbitrary units) of each A-band was quantified by densitometry using a GS710 Calibrated imaging Densitometer (Bio-Rad Laboratories, Gladesville, New South Wales, Australia). The mean ± SEM OD A-MMP-2 or A-MMP-9 was then measured and expressed as a fold-change of the mean value obtained from the NDW control group (which was expressed as 1).

### Assessment of kidney function

At 8-weeks post-HS loading, all mice were individually housed in metabolic cages for 24-h (hr) urine collection. The urinary protein level (as a measure of proteinuria) was determined for each sample using the Bradford protein assay (Bio-Rad Protein Assay Dye Reagent Concentration; Gladesville, NSW, Australia) [41]. Urinary and plasma creatinine concentrations were measured using Creatinine assay kits (ab65340; Abcam, Cambridge, UK) according to the manufacturer’s instructions, and were used to derive creatinine clearance using the following formula described previously [41]:

: $$\frac{{{\text{Urine }}\;{\text{creatinine }}\;{\text{concentration }}\left( {{\text{nmol}}/{\text{mL}}} \right) \, \times {\text{ urine}}\;{\text{ volume }}\left( {{\text{mL}}/{\text{24hr}}} \right)}}{{{\text{Plasma creatinine concentration }}\left( {{\text{nmol}}/{\text{mL}}} \right)}}$$.

## Statistical analysis

Data were expressed as the mean ± SEM unless otherwise stated and were statistically analysed using GraphPad Prism (v9.3.0; GraphPad Software Inc, San Diego, CA, USA). SBP was analysed with a two-way analysis of variance (ANOVA; treatment × time), while all the other data were analysed with a one-way ANOVA. Post hoc tests were only conducted if the overall ANOVA p-value achieved statistical significance (i.e., p < p0.05) and there was no significant variance in homogeneity. A Tukey’s post-hoc test was conducted to allow for multiple comparisons between groups. Non-parametric (Kruskal–Wallis) tests were conducted for data that were graded based on a scoring system (i.e. inflammation score, tubular abnormalities, protein cast formation, glomerulosclerosis); or normalised to the control group (i.e. Relative OD A-MMP-2 and A-MMP-9). All data were included unless any values were > 2 standard deviations (SDs) from the mean. In the case of A-MMP-2 and A-MMP-9 levels, n = 6 samples per group were analysed. Differences were considered statistically significant at p < 0.05, and the threshold value was not varied during the study.

## Results

### Generation and characterisation of engineered BM-MSCs

Human BM-MSCs were transduced with a lentivirus containing human relaxin-2 cDNA (RLX) and green fluorescent protein (GFP), under a eukaryotic translation elongation factor-1α promoter (termed BM-MSCs-eRLX + GFP; Fig. 1A) or GFP alone (BM-MSCs-eGFP). These BM-MSCs-eRLX + GFP and BM-MSCs-eGFP were characterised to confirm their cellular identity using the minimum criteria established by the International Society for Cellular Therapy [44]. Flow cytometry revealed transduction efficiencies of 72.4% and 94.4% at a multiplicity of infection of 2, for passage 4 (P4) BM-MSCs-eRLX + GFP and BM-MSCs-eGFP, respectively, from which both showed spindled-shaped morphology and expression of GFP (in 77.5% and 86.7% of P6 BM-MSCs-eRLX + GFP and BM-MSCs-eGFP, respectively; Fig. 1B). BM-MSCs-eRLX + GFP expressed RXFP1 (Fig. 2A) on their cell surface and were able to functionally differentiate into adipocytes, osteocytes and chondrocytes as evidenced by positive histological staining with Oil-red-O, Alizarin red and alcian blue, respectively; or immunohistochemical staining for fatty acid binding protein 4 (FABP4), osteocalcin and aggrecan, respectively (Fig. 2B).

Compared to the proliferation and migration capacity of 1 × 10^6^ naïve BM-MSCs in culture, 1 × 10^6^ BM-MSCs stimulated with either 1 ng/ml or 10 ng/ml RLX, or 1 × 10^6^ BM-MSCs-eRLX + GFP or BM-MSC-eGFP did not undergo any further proliferation after 3-days (Fig. 2C) or migration after 24-h (Fig. 2D). However, 1 × 10^6^ BM-MSCs-eRLX + GFP did undergo significantly increased proliferation (by ~ 18%) after 7-days (Fig. 2C), and produced 29 ± 3 ng/ml and 31 ± 3 ng/ml RLX in the supernatant after 3- and 7-days of culture, respectively (Fig. 2E). These RLX levels had previously been shown to attenuate TGF-β1-induced human myofibroblast-induced collagen production and promote myofibroblast-mediated collagenase activity in vitro [45–47]. Detectable levels of RLX were also measured from the exosomes that were isolated and lysed from BM-MSCs-eRLX + GFP (Figure S1 [Supplementary File]) that were cultured for 3-days (~ 181.4 pg/ml), whereas the conditioned media from the exosome extraction contained ~ 2.3 ng/ml of RLX. This suggested that the RLX secreted from BM-MSCs-eRLX + GFP was predominantly secreted by the cells as a soluble peptide rather than being packaged within BM-MSC-derived exosomes.

### Protein secretion profile of BM-MSCs-eRLX + GFP

After 7-days of culture, BM-MSCs-eRLX + GFP secreted an enhanced but finely tuned immunomodulatory, angiogenic and ECM-remodelling/cell proliferative capacity compared to naïve BM-MSCs (Fig. 2F). The increased secretion of monocyte chemoattractant protein (MCP)-3 (by ~ 43%) and interleukin (IL)-8 (by ~ 88%) by BM-MSCs-eRLX + GFP may have been linked to the immunomodulatory effects of these cells in regulating macrophage and neutrophil trafficking, respectively. In a complementary manner, BM-MSCs-eRLX + GFP released lower levels of MCP-1 (by ~ 14%) and IL-6 (by ~ 21%) compared to naïve BM-MSCs, which may have been related to their anti-inflammatory effects. Regarding ECM remodelling, BM-MSCs-eRLX + GFP secreted less pro-fibrotic mediators such as insulin-like growth factor binding protein (IGFBP)-2 (by ~ 50%), IGFBP-3 (by ~ 11%), serpin E1, urokinase-type plasminogen activation receptor (uPAR) and platelet-derived growth factor containing two A subunits (PDGF-AA) (by ~ 13%-28%), coupled with higher levels of CD147 (inducer of MMPs; by ~ 31%). Furthermore, BM-MSCs-eRLX + GFP showed heightened secretion of tissue-reparative factors including angiopoietin-1 (by ~ tenfold), endoglin (by ~ 49%) and angiogenin (by ~ 28%), and produced a significantly higher amount of RLX compared to naïve BM-MSCs. Importantly, several tumorigenic-related factors were down-regulated by BM-MSCs-eRLX + GFP, such as IGFBP2, vascular endothelial growth factor (VEGF) and uPAR, compared to that released by naïve BM-MSCs, which pointed to the potential safety of utilising the therapeutic impact of these cells.

### BM-MSCs-eRLX + GFP homed to the kidneys of IRI and HS-fed mice

**Fig. 3:**
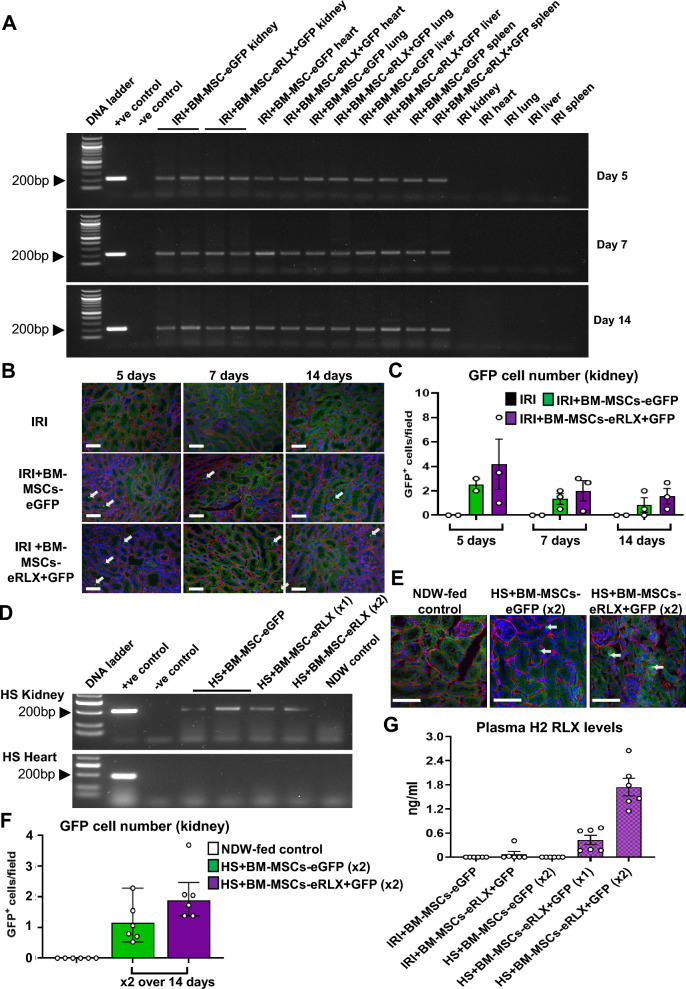
Confirmation that i.v-injected BM-MSCs-eRLX + GFP homed to the injured kidney of IRI and HS-fed mice post-transplantation. **A** Shown are representative images of SYBR Safe-stained 1.5% agarose gels that indicate a PCR product corresponding to *EGFP* (at the expected size of 196 bp) from either 1 × 10^6^ cultured BM-MSCs-eGFP that were not injected into mice (positive (+ ve) control) or the kidneys, hearts, lungs, livers and spleens of IRI mice at 5-days, 7-days and 14-days post-a single injection of BM-MSCs-eGFP or BM-MSCs-eRLX + GFP; or **D** from the kidney and heart of HS fed-mice 7-days after the second injection of BM-MSCs-eGFP (× 2) or BM-MSCs-eRLX + GFP (× 2) that were once-weekly injected at weeks-7 and -8 post-HS feeding or 14-days after a single-i.v-injection of BM-MSCs-eRLX + GFP (× 1) that was administered at the beginning of week-7 post-HS feeding. The full-length blots of panel **A** and **D** are shown in Supplementary Fig. 4 and Supplementary Fig. 5, respectively. **A**, **D** This PCR product was not found in the negative (-ve) control reaction, in which distilled water (dH_2_O) replaced cDNA, or from the genomic DNA extracted from **A** these organs of IRI mice that were not injected with any cells; or **D** from the DNA extracted from the kidneys or heart of NDW-fed control mice. In each case, a **A** 100 bp or **D** 50 bp DNA ladder was included. **B**, **E** GFP-labelled cells (indicated by the white arrows) were also detected by fluorescence microscopy and were predominantly co-localised with DAPI-stained cells within the tubules of the left kidney of **B** IRI mice or **E** HS-fed mice that were injected with BM-MSCs-eGFP or BM-MSCs-eRLX + GFP, but not in **B** untreated IRI control or **E** NDW-fed control mice. The basement membrane structure (collagen IV) was outlined by red fluorescence. **C**, **F** Also shown is the mean ± SEM of the quantification of GFP^+^ cells/field from 6 non-overlapping fields each sample at × 200 magnification; from (**C**) n = 2–3 mice per time-point per group from the IRI model; and **F** n = 6 mice per group from the HS model. Scale bar = 100 µm in each case. **G** Additionally shown is the mean ± SEM plasma H2 RLX levels from each group shown; from n = 6 mice per group

EGFP gene expression (expected size 196 bp) was identified by PCR analysis in the kidneys of IRI mice, 5-, 7- and 14-days after a single i.v-injection of BM-MSCs-eRLX + GFP (Fig. 3A-C); and in HS-fed mice, 7-days after a once-weekly i.v injection of BM-MSCs-eRLX + GFP (× 2), or 14-days after a single i.v-injection of BM-MSCs-eRLX + GFP (× 1), at 8-weeks post-HS loading (Fig. 3D-F). The *EGFP* expression identified in the kidneys of these animals corresponded to the same product that was derived from 1 × 10^6^ BM-MSC-eGFPs that were not injected into mice (positive control). EGFP gene expression was also identified in other organs of IRI mice such as the lungs, heart, spleen and liver 5–14-days after a single injection of cells (potentially owing to the transportation of cells to these organs during the reperfusion phase in which there is an increase in blood flow; Fig. 3A), but was not identified in the heart of HS-fed mice (Fig. 3D). No *EGFP* expression, however, was detected in the kidneys of sham or normal drinking water (NDW)-fed controls or when distilled water replaced cDNA (as negative controls). Furthermore, GFP-labelled cells were detected by fluorescence microscopy and predominantly localised to DAPI-nuclear stained cells within the renal tubular regions of IRI mice that were injected once with BM-MSCs-eGFP or BM-MSCs-eRLX + GFP (Fig. 3B) or HS-fed mice that were injected once-weekly with BM-MSCs-eGFP or BM-MSCs-eRLX + GFP (Fig. 3E), but not NDW-fed mice (negative control). Quantitative analysis of these GFP-labelled cells revealed a trend towards an increase in the number of GFP^+^ cells that had infiltrated the kidney of IRI + BM-MSC-eRLX + GFP-treated mice after 5-days (by ~ 87%) and 7-days (by ~ 54%) post-administration (Fig. 3C), compared to their BM-MSC-eGFP-treated counterparts. Similarly, ~ 74% more GFP^+^ cells were detected in the kidneys of HS + BM-MSCs-eRLX + GFP (× 2)-treated mice compared to their BM-MSCs-eGFP (× 2)-treated counterparts (p = 0.08 vs the BM-MSCs-eGFP-treated group; Fig. 3F).

Additionally, circulating RLX levels were detected in IRI mice treated with BM-MSCs-eRLX + GFP (0.07 ± 0.05 ng/ml; Fig. 3G) by 7-days post-cell administration. Comparatively, plasma RLX levels were slightly higher in HS + BM-MSCs-eRLX + GFP-treated mice, 14-days after a single i.v-injection of cells (0.4 ± 0.1 ng/ml) or 7-days after the second injection of cells (1.7 ± 0.2 ng/ml), which mirrored circulating RLX levels found in pregnant women [48] and in human subjects enrolled in various clinical trials who received 25–100 µg/kg/day of RLX [49].

### BM-MSCs-eRLX + GFP attenuated IRI-induced kidney damage and fibrosis

**Fig. 4:**
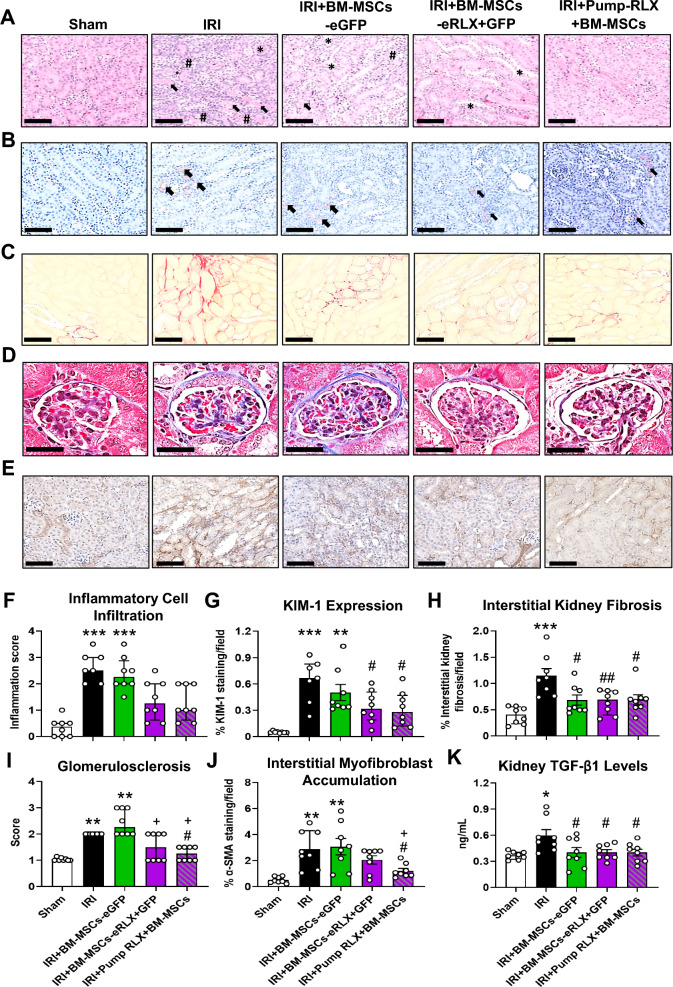
The effects of IRI and the treatments evaluated on kidney inflammation, tubular damage and fibrosis. **A**, **B** Representatives images of **A** H&E-stained and **B** KIM-1 immunohistochemically stained kidney sections (taken at × 400 magnification) show the extent of **A** kidney structural changes, protein cast formation (arrow), tubular dilation (*) as well as inflammatory cell aggregates (#); and **B** KIM-1 (brown staining)-associated tubular epithelial damage, respectively, in each group studied. **C-E** Representative images of **C** picrosirius red-stained, **D** Masson’s trichrome-stained and **E** α-SMA immunohistochemically-stained kidney sections (at × 400 magnification) show the extent of **C** red-stained tubulointerstitial and **D** blue-stained glomerular matrix deposition, as well as **E** brown-stained interstitial myofibroblast accumulation in each of the groups evaluated, respectively; from n = 7–8 mice per group. Scale bar = 100 µm in each case. **F-K** Also shown is the semi-quantification of the **F** median ± IQR inflammatory cell infiltration within the kidney; or the mean ± SEM (**G**) KIM-1 staining per field analysed; **H** % interstitial kidney fibrosis staining per field analysed; **J** % α-SMA staining per field analysed; or **K** TGF-β1 expression levels (determined by ELISA analysis of kidney protein extracts), as well as the **I** median ± IQR glomerulosclerosis score; from n = 8 non-overlapping fields of view or 30 glomeruli per section analysed; from n = 7–8 mice per group. *p < 0.05, **p < 0.01, ***p < 0.001 vs the sham-operated group; ^#^p < 0.05, ^##^p < 0.01 vs the IRI alone group; ^+^p < 0.05 vs the IRI + BM-MSC-eGFP group; as determined using **G**, **H**, **J**, **K** a one-way ANOVA and Tukey’s *post-hoc* test or **F**, **I** a non-parametric Kruskal–Wallis test with Dunn’s *post-hoc* analysis

Compared to sham-operated controls, IRI mice maintained similar body weight (BW), kidney weight (KW) and KW:BW ratio (STable 3 [Supplementary File]), but underwent a significant increase in kidney inflammation, structural damage (by 5–6.5-fold; Fig. 4A and F) and KIM-1-associated tubular damage (by 10.6-fold; Fig. 4B and G) by 7-days post-injury. Whilst each of these elevated measures were unaffected by BM-MSCs-eGFP treatment alone, BM-MSCs-eRLX + GFP treatment attenuated the IRI-induced increase in kidney inflammation and structural damage (by ~ 50–55%; Fig. 4F) and significantly prevented the KIM-1-stained tubular damage (Fig. 4G), to levels that were not different to that measured in sham controls. In each case, this was to an equivalent extent as Pump-RLX + BM-MSC treatment administered separately.

IRI mice also presented with significantly increased interstitial kidney fibrosis (by 2.1-fold; Fig. 4C and H), glomerulosclerosis (by one-fold; Fig. 4D and I), interstitial myofibroblast accumulation (by 4.5-fold; Fig. 4E and J) and TGF-β1 levels (by 0.6-fold; Fig. 4K) at 7-day post-injury, compared to that measured in sham controls. BM-MSCs-eGFP treatment only effectively suppressed the IRI-induced interstitial kidney fibrosis (Fig. 4H) and TGF-β1 levels (Fig. 4K) after 7-days of treatment, but did not affect the other measures of fibrosis evaluated. In comparison, BM-MSCs-eRLX + GFP treatment equivalently prevented these measures in addition to the IRI-induced glomerulosclerosis (Fig. 4I), and also attenuated the IRI-induced interstitial myofibroblast accumulation (by 33%; Fig. 4J), to levels that were not different to that measured in sham-operated controls. In each case, this was to an equivalent extent as Pump-RLX + BM-MSC treatment administered separately. These findings demonstrated the broader renoprotection offered by BM-MSCs-eRLX + GFP over BM-MSCs-eGFP against IRI.

### BM-MSCs-eRLX + GFP therapeutically reduced HS-induced hypertension and renal vascular rarefaction

**Fig. 5:**
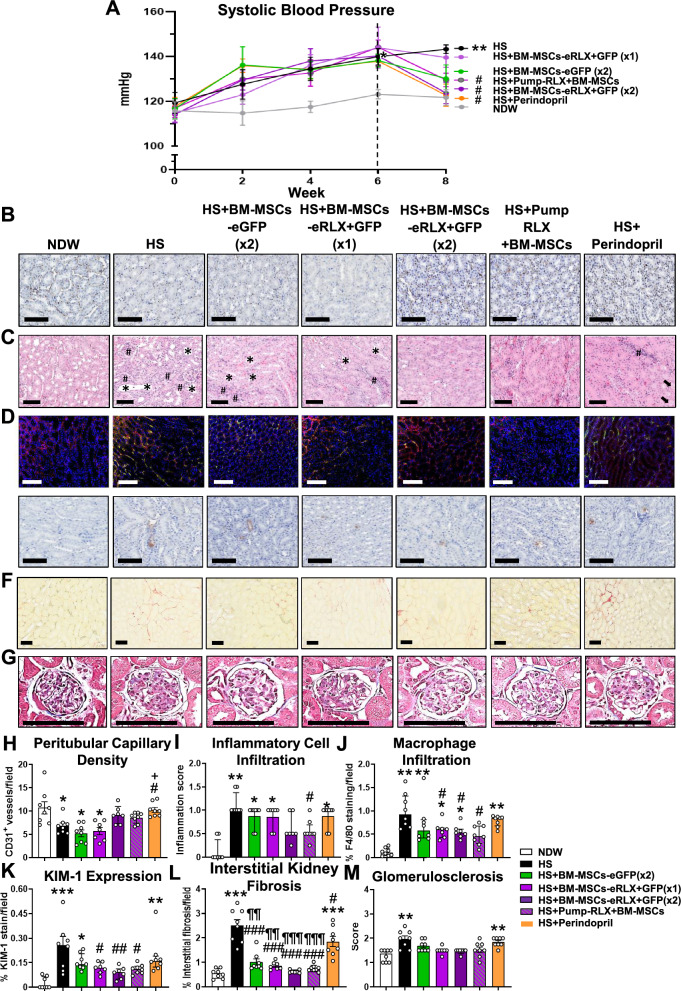
The effects of HS and the treatments evaluated on systolic blood pressure (SBP), kidney endothelial and tubular epithelial damage, kidney inflammation and fibrosis. **A** Shown is the mean ± SEM SBP, from week-0 to week-8 of the HS-induced model established and treated; from n = 8 mice per group. **B-G** Representatives images of **B** CD31^+^ immunostaining, **C** H&E-stained inflammatory cell infiltration, **D** F4/80-stained immunofluorescence, **E** KIM-1 immunostaining, **F** picrosirius red-stained and **G** Masson’s trichrome-stained kidney sections (taken at × 400 magnification) show the extent of **B** peritubular capillary density; **C** kidney structural changes, protein cast formation (arrow), tubular dilation (*) as well as inflammatory cell aggregates (#); **D** macrophage (green-staining) infiltration in sections that were counterstained with collagen IV (red-staining); **E** KIM-1 (brown staining)-associated tubular epithelial damage; **F** red-stained tubulointerstitial; and **G** blue-stained glomerular matrix deposition within the kidney in each group studied. Scale bar = 100 µm in each case. **H-M** Also shown is the semi-quantification of **H** CD31^+^ capillary density/field; **I** inflammatory cell; and **J** macrophage infiltration within the kidney; **K** kidney proximal tubular damage; **L** interstitial kidney fibrosis; or **M** glomerulosclerosis, expressed as **H**, **I**, **J**, **L**, **M** mean ± SEM or **K** median ± IQR per field analysed, from n = 8 non-overlapping fields of view; from n = 7–8 mice per group. *p < 0.05, **p < 0.01, ***p < 0.001 vs the NDW control group; ^#^p < 0.05, ^##^p < 0.01, ^###^p < 0.001 vs the HS alone group; ^+^p < 0.05 vs the HS + BM-MSC-eGFP group; ^¶¶^p < 0.01, ^¶¶¶^p < 0.001 vs the HS + Perindopril-treated group; as determined using **H**, **J**, **K**, **L** a one-way ANOVA and Tukey’s *post-hoc* test or **I**, **M** a non-parametric Kruskal–Wallis test with Dunn’s *post-hoc* analysis

Compared to normal drinking water (NDW)-fed mice, HS (2% NaCl)-fed mice underwent a 10–15% loss of BW, in the absence of any marked changes in KW or KW:BW ratio (STable 3 [Supplementary File]), and a progressive increase in SBP, which reached ~ 20 mmHg above baseline measurements after8-weeks (Fig. 5A). All treatments blunted the HS-induced loss of BW when administered from weeks-7–8, towards that of NDW-fed mice. Furthermore, when administered once-weekly from weeks-7–8 (× 2), BM-MSCs-eGFP blunted (by 11-12 mmHg), whilst BM-MSCs-eRLX + GFP or Pump-RLX + BM-MSCs significantly normalised the HS-induced increase in SBP, to the same extent as perindopril treatment (Fig. 5A). In line with an elevated BP triggering renal vascular endothelial damage and dysfunction [50], HS-fed mice had significantly reduced kidney expression of CD31^+^ peritubular capillary density after 8-weeks (by ~ 35–40%; Fig. 5B and H) compared to their NDW-fed controls. Consistent with the treatment-effects on SBP, the HS-induced loss of perivascular capillary density was unaffected by BM-MSCs-eGFP (× 2) treatment, but was restored to levels measured in NDW-fed mice by BM-MSCs-eRLX + GFP (× 2), Pump-RLX + BM-MSCs or perindopril treatment (Fig. 5B and H).

### BM-MSCs-eRLX + GFP therapeutically reduced the HS-induced kidney inflammation, tubular damage and fibrosis

**Fig. 6:**
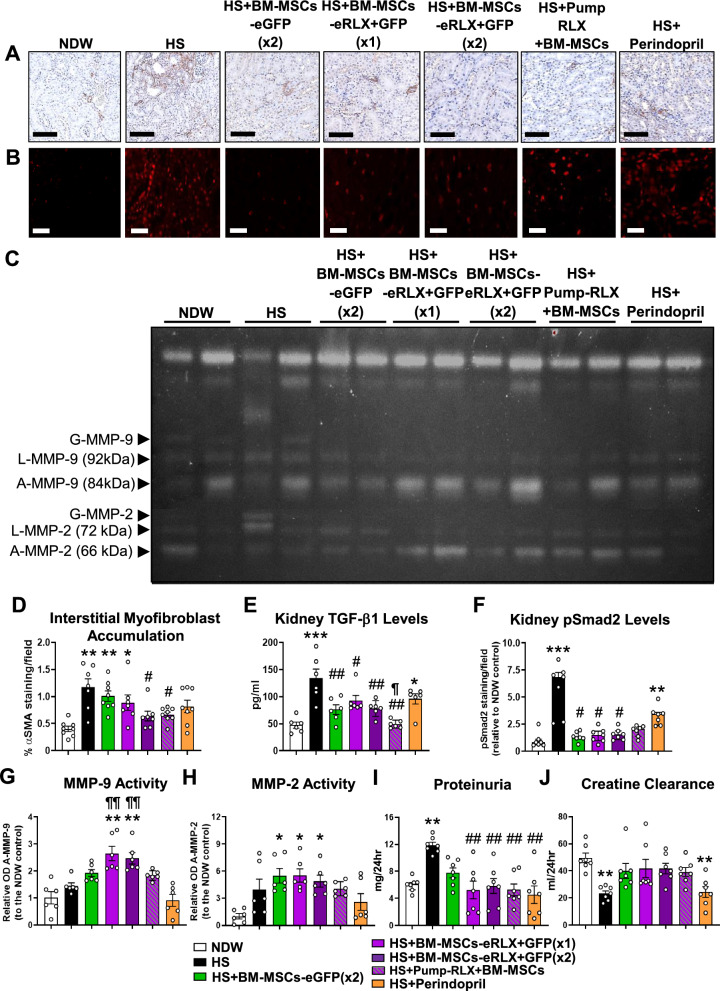
The effects of HS and the treatments evaluated on additional measures that control the rate of kidney fibrosis and on kidney function. **A**, **B** Representative images of **A** α-SMA immunostaining and **B** pSmad2 immunofluorescence staining within kidney sections (at × 400 magnification) show the extent of **A** brown-stained interstitial myofibroblast accumulation and; **B** red-stained pSmad2 expression in each of the groups evaluated, respectively. Scale bar = **A** 100 µm or **B** 50 µm. **C** A representative gelatin zymography shows the extent of glycosylated (G), latent (L) and active (A) MMP-9 (gelatinase B) and MMP-2 (gelatinase A) levels within the kidneys of each of the groups evaluated. An additional n = 4 samples per group were run on separate zymographs, which produced similar results. **D-F** Also shown are the mean ± SEM % **D** α-SMA staining/field; **E** TGF-β1 expression levels (determined by ELISA analysis of kidney protein extracts); or **F** % pSmad2 staining/field (relative to that in the NDW control group), from n = 8 non-overlapping fields of view or 30 glomeruli per section analysed; from n = 6–8 mice per group–for the analysis of tubulointerstitial fibrosis, glomerular matrix deposition, interstitial myofibroblast accumulation and pSmad2 staining. **G-J** Additionally shown are the mean ± SEM **G** relative OD A-MMP-9; **H** relative OD A-MMP-2; **I** proteinuria; or **J** creatine clearance; from n = 6–7 mice per group. *p < 0.05, **p < 0.01, ***p < 0.001 vs the NDW control group; ^#^p < 0.05, ^##^p < 0.01 vs the HS alone group; ^¶¶^p < 0.01 vs the HS + Perindopril-treated group; as determined **D**, **E**, **F**, **I**, **J** using a one-way ANOVA and Tukey’s *post-hoc* test or **G**, **H** a non-parametric Kruskal–Wallis test with Dunn’s *post-hoc* analysis

Compared to NDW-fed controls, HS-fed mice exhibited interstitial matrix expansion and significantly increased kidney inflammation (Fig. 5C and I), which consisted of F4/80^+^ macrophage (by 7–8-fold; Fig. 5D and J), CD45^+^F4/80^+^CD206^−^ M1-like macrophage, CD45^+^F4/80^+^CD206^+^ M2-like macrophage and CD45^+^CD11^+^ dendritic cell (Figure S2 [Supplementary File]) infiltration. HS-fed mice also presented with tubular KIM-1-associated damage (by 8.7-fold; Fig. 5E and K); pro-inflammatory monocyte chemoattractant protein-1 (MCP-1; by 1.6-fold), tumour necrosis factor (TNF)-α (by 1.8-fold) and interleukin (IL)-1β (by ten-fold) (Figure S3 [Supplementary File]) levels; interstitial kidney fibrosis (by 3.7-fold; Fig. 5F and L); glomerular matrix accumulation (by 0.5-fold; Fig. 5G and M); interstitial myofibroblast accumulation (by 1.9-fold; Fig. 6A and D); kidney TGF-β1 expression (by 1.8-fold; Fig. 6E); phosphorylated Smad2 (pSmad2) expression (by eight-fold; Fig. 6B and F); and matrix metalloproteinase (MMP)-2 activity (by 2.9-fold; Fig. 6C and H) after 8-weeks.

BM-MSC-eGFP (× 2) treatment significantly reduced the HS-induced TNF-α levels (Figure S3 [Supplementary File]), interstitial kidney fibrosis (Fig. 5L), TGF-β1(Fig. 6E) and pSmad2 (Fig. 6F) levels, attenuated the HS-induced glomerulosclerosis to levels that were not different to that measured in NDW-fed controls (Fig. 5M), and increased MMP-2 activity (Fig. 6C and H) after 2-weeks of treatment. However, these cells failed to affect the HS-induced kidney inflammation (Fig. 5I) including macrophage infiltration (Fig. 5J), KIM-1-associated tubular damage (Fig. 5K), interstitial myofibroblast accumulation (Fig. 6D) or MMP-9 activity (Fig. 6C and G). On the other hand, all these inflammatory measures including M1-like macrophage infiltration, dendritic cell infiltration (Figure S2 [Supplementary File]), kidney MCP-1 and TNF-α levels (Figure S3 [Supplementary File]), as well as measures of fibrosis were significantly reduced or attenuated by BM-MSC-eRLX + GFP treatment. In each case, this was to an equivalent extent as Pump-RLX + BM-MSC treatment administered separately. However, only BM-MSC-eRLX + GFP treatment also significantly promoted both collagen-degrading MMP-9 (Fig. 6G) and MMP-2 (Fig. 6H) activity in HS-fed mice.

Notably, i) a single injection of BM-MSCs-eRLX + GFP (× 1) ameliorated the HS-induced KIM-1-associated tubular damage, TNF-α, interstitial fibrosis, glomerulosclerosis and pSmad2 levels, and significantly promoted MMP-9 and MMP-2 activity to an equivalent extent as once-weekly administration of BM-MSCs-eRLX + GFP (× 2), 14-days after being injected; and ii) all BM-MSC-based therapies reduced the HS-induced interstitial kidney fibrosis to a greater extent than perindopril (Fig. 5L). This was likely due to the greater ability of all BM-MSC-based therapies to promote gelatinase activity (Fig. 6G and H) compared to perindopril.

### BM-MSCs-eRLX + GFP therapeutically restored the HS-induced kidney functional impairment

HS-fed mice exhibited significantly increased proteinuria (by one-fold; Fig. 6I) but reduced creatinine clearance (by 0.5-fold; Fig. 6J) after 8-weeks. Both measures of kidney impairment were only significantly ameliorated by BM-MSCs-eRLX + GFP or Pump-RLX + BM-MSC treatment, but not by BM-MSCs-eGFP nor perindopril treatment. Once again, these collective findings demonstrated the broader renoprotection offered by BM-MSCs-eRLX + GFP over BM-MSCs-eGFP or ACE inhibition against HS-induced nephropathy.

### BM-MSCs-eRLX + GFP maintained their broad therapeutic efficacy in the presence of ACE inhibition

**Fig. 7:**
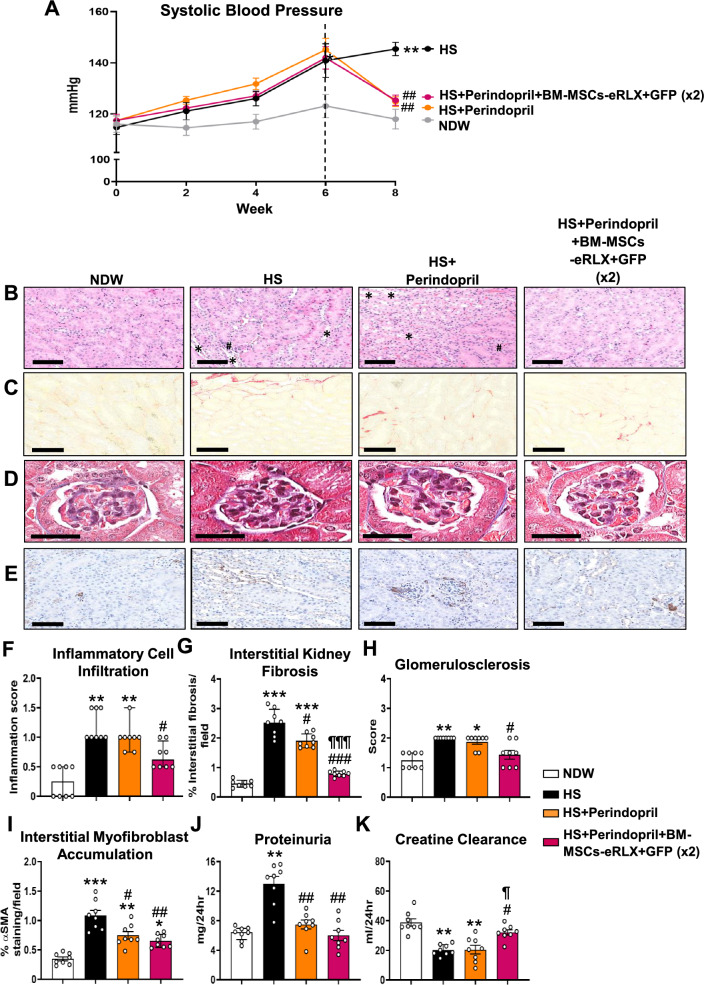
BM-MSCs-eRLX + GFP maintained their therapeutic efficacy in the presence of perindopril (ACEi). (A) Shown is the mean ± SEM SBP, from week-0 to week-8 of the HS-induced model established and treated; from n = 8 mice per group shown. **B-E** Representatives images of **B** H&E-stained inflammatory cell infiltration, **C** picrosirius red-staining, **D** Masson’s trichrome-staining, and **E** α-SMA immunostaining (taken at × 400 magnification) show the extent of **B** kidney structural changes, tubular dilation (*) as well as inflammatory cell aggregates (#); **C** red-stained tubulointerstitial fibrosis; **D** blue-stained glomerular matrix deposition; and **E** brown-stained interstitial myofibroblast accumulation in each of the groups evaluated, respectively. Scale bar = 100 µm in each case. **F-K** Also shown is the median ± IQR **F** inflammatory cell infiltration; or (H) glomerulosclerosis score; or the mean ± SEM **G** % interstitial kidney fibrosis staining per field analysed; **I** % α-SMA staining/field; **J** proteinuria (mg/24 h); or **K** creatinine clearance (ml/24 h); from n = 7–8 mice per group. *p < 0.05, **p < 0.01, ***p < 0.001 vs the NDW control group; ^#^p < 0.05, ^##^p < 0.01; ^###^p < 0.001 vs the HS alone group; ^¶^p < 0.05, ^¶¶¶^p < 0.001 vs the HS + Perindopril-treated group; as determined using **G**, **I**, **J**, **K** a one-way ANOVA and Tukey’s *post-hoc* test or **F**, **H** a non-parametric Kruskal–Wallis test with Dunn’s *post-hoc* analysis

It was also determined if BM-MSC-eRLX + GFP could maintain their therapeutic efficacy (as an adjunct therapy) in the presence of a frontline treatment for CKD (such as perindopril), when administered to a separate cohort of HS-fed mice. In line with the findings presented in Fig. 5 and Fig. 6, perindopril treatment abrogated the HS-induced increase in SBP (Fig. 7A) and proteinuria (Fig. 7J), and partially reduced interstitial kidney fibrosis (by ~ 30%; Fig. 7C and G) and myofibroblast accumulation (by ~ 45%; Fig. 7E and I) after 2-weeks, but failed to affect the HS-induced kidney inflammation (Fig. 7B and F), glomerulosclerosis (Fig. 7D and H) or the loss of creatine clearance (Fig. 7K). Comparatively, perindopril and BM-MSCs-eRLX + GFP treatment of HS-fed mice normalised the HS-induced increase in SBP to an equivalent extent as perindopril treatment alone, but significantly reduced all measures of kidney inflammation, fibrosis and function evaluated to levels measured in NDW-fed mice. Notably, the combined effects of perindopril and BM-MSCs-eRLX + GFP reduced the HS-induced interstitial kidney fibrosis (Fig. 7G) and restored the impairment of creatine clearance (Fig. 7K) to a significantly greater extent than perindopril treatment alone; confirming that BM-MSCs-eRLX + GFP could maintain their broad renoprotective effects in the presence of ACE inhibition.

### Evaluation of the long-term safety and anti-fibrotic efficacy of BM-MSCs-eRLX + GFP

**Fig. 8:**
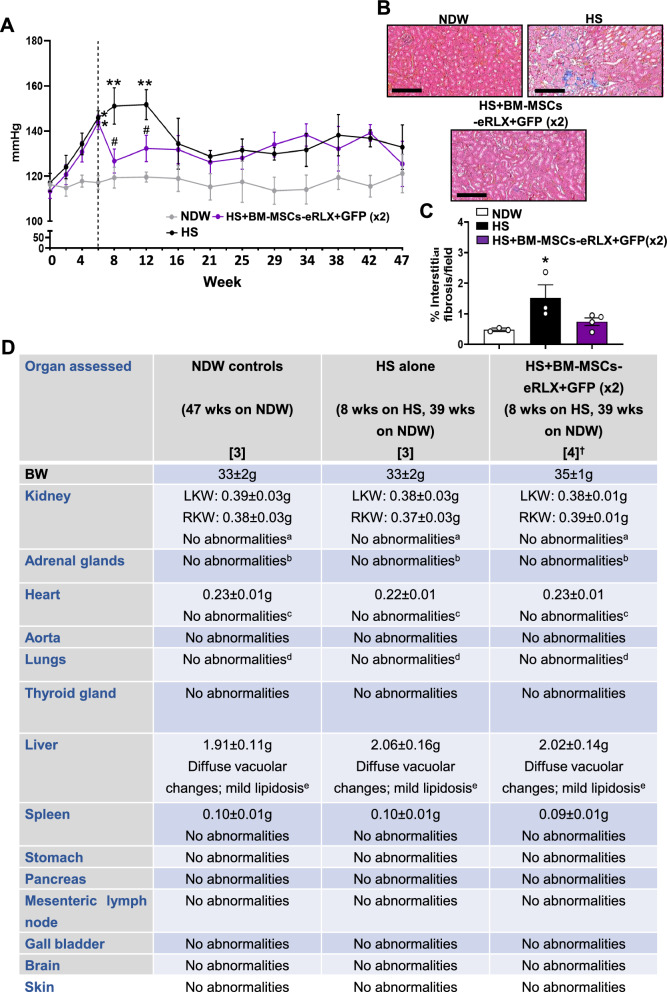
Evaluation of the long-term efficacy and safety of BM-MSCs-eRLX + GFP. **A** Shown is the mean ± SEM SBP, as measured once-fortnightly from week-0 to week-8, and then once-monthly from week-8 to week-47, from the HS-induced model established and treated; from NDW (n = 3) or HS (n = 3)-fed mice, and HS + BM-MSCs-eRLX + GFP (× 2)-treated mice (n = 4). **B** Representative images of Masson’s trichrome-staining show the extent of blue-stained tubulointerstitial fibrosis in each of the groups studied. Scale bar = 200 µm. **C** Also shown is the mean ± SEM % interstitial kidney fibrosis staining per field analysed from each group evaluated; from n = 8 non-overlapping fields of view analysed; from n = 3–4 mice per group. *p < 0.05, **p < 0.01 vs the NDW control group; ^#^p < 0.05vs the HS alone group; as determined using a two-way ANOVA (A) or one-way ANOVA (C) and Tukey’s *post-hoc* test in each case. **D** A summary of the pathological assessment of the three groups of mice evaluated, 9 months after BM-MSCs-eRLX + GFP (× 2) had stopped. ^†^n = 2 out of 6 HS-fed mice injected with BM-MSC-eRLX + GFP (× 2) started to chew their tails after the i.v-injection of cells, and had to be humanely killed shortly thereafter–so could not be assessed. ‘No abnormalities’ implies that no significant abnormalities were detected in these mice. However: ^a^n = 1 HS-fed mouse and n = 2 HS + BM-MSCs-eRLX + GFP-treated mice had mild multifocal interstitial lymphoplasmacytic infiltrations, and n = 1 HS + BM-MSCs-eRLX + GFP-treated mouse had mild multifocal cystically dilated renal tubules and mild early renal hydronephrosis; ^b^all mice had evidence of subcapsular spindle cell hyperplasia (adrenal gland); ^c^several mice from each group had focal to multifocal mineralisation of the right ventricle; ^d^several mice from each of the HS-fed groups had focal bronchoalveolar adenoma (lung); ^e^almost all mice had age-related diffuse glycogen-type vacuolar changes and mild, diffuse centrilobular hepatocellular lipidosis. The organ weights presented are expressed as the mean ± SEM; from n = 3–4 mice per group (as denoted by the numbers in the square brackets)

As the FDA requires that the safety of any cell-based therapy is assessed 6-to-9 months after treatment cessation, the long-term safety and renal anti-fibrotic efficacy of BM-MSCs-eRLX + GFP was assessed in HS-fed mice 9-months after treatment cessation. HS-fed mice exhibited a gradual elevation in SBP (Fig. 8A), reaching ~ 25-30 mmHg above baseline measurements taken after 6–12-weeks. These hypertensive mice had interstitial kidney fibrosis levels (0.9–2.4% per field; Fig. 7B and C) that had moderately resolved compared to what was measured at 8 weeks post-HS consumption (Fig. 5L), but were still significantly higher than that measured in their NDW-fed counterparts (0.4–0.6% per field). Notably, this HS-induced rise in SBP partially resolved (by ~ 15-20 mmHg) by 16–47-weeks post-HS discontinuation, but remained at ~ 15-20 mmHg above respective measurements taken at baseline during this interval. The HS-induced increase in SBP was significantly reduced by BM-MSCs-eRLX + GFP after 2-to-6 weeks of treatment. However, SBP levels in the BM-MSCs-eRLX + GFP-treated group remained similar to that of their HS-fed counterparts from weeks 16–47 post-treatment cessation. Remarkably, interstitial kidney fibrosis levels were still suppressed in the BM-MSCs-eRLX + GFP-treated group (0.4–1.0% per field) after 9-months of treatment cessation, and were not different to that measured from NDW-fed mice (Fig. 8C).

NDW-fed mice did not have any significant pathological abnormalities at 59-weeks of age, but presented with an age-induced subcapsular spindle cell hyperplasia (adrenal gland), diffuse vacuolar changes and centrilobar hepatocellular lipidosis (liver) (Fig. 8D). Similarly, no major abnormalities were observed in age-matched HS-fed mice and HS-fed mice treated BM-MSCs-eRLX + GFP (× 2). However, these mice presented with the same minor abnormalities that were detected in NDW-fed control mice, and *n* = 2 BM-MSCs-eRLX + GFP (× 2)-treated mice had mild multifocal interstitial lymphoplasmacytic infiltrations, whilst n = 1 of these two BM-MSCs-eRLX + GFP (× 2)-treated mice also had mild multifocal cystically dilated renal tubules and mild early hydronephrosis (kidney).

## Discussion

For the first time, a lentiviral vector that contained an expression cassette for an anti-fibrotic hormone, relaxin (RLX) [6, 16] and GFP (as a fluorescent reporter), was stably used to transduce human BM-MSCs. Remarkably, our data highlighted that these BM-MSCs-eRLX + GFP produced biologically active and therapeutic RLX levels in vitro (~ 30 ng/ml after 3–7-days in culture) and in vivo (~ 1-2 ng/ml after once-weekly injections over 2-weeks), and retained their differentiation and proliferative capacity compared to naïve BM-MSCs in vitro. In experimental models of IRI-induced AKI or HS-loading induced CKD, BM-MSCs-eRLX + GFP could be identified in the kidneys up to 7–14-days post-transplantation, with a trend towards improved engraftment capacity compared to naïve BM-MSCs. This enhanced viability and engraftment of BM-MSCs-eRLX + GFP to the injured site by the presence of RLX, in turn, prolonged the anti-fibrotic properties of RLX (which has a short in vivo half-life of ~ 4–8 h [31, 32]) within the injured kidney for up to 14-days following a single injection.

Interestingly, both BM-MSCs-eGFP and BM-MSCs-eRLX GFP were found to be widely distributed in the injured kidney, heart lung, spleen and liver of IRI mice from 5-to-14-days post-transplantation, but were selectively found in the kidneys of HS-fed mice from 7-to-14-days post-administration. These findings extended our previous findings in IRI mice [34], which found that BM-MSCs-eGFP migrated to the injured kidney within 24 h of transplantation and remained in the kidney after 72 h. This differential homing and distribution of the i.v-injected MSCs could be impacted by various factors related to the specific pathophysiology, inflammatory response to injury and microenvironment of the different models studied, but are consistent with previous reports of the biodistribution of MSCs in murine models of acute kidney injury [51, 52]. Notably though, whilst BM-MSCs-eGFP demonstrated anti-hypertensive and anti-fibrotic effects in the preclinical models established, it was clearly evident that the added effects of RLX with BM-MSCs provided broader (anti-inflammatory, anti-remodelling and tissue-reparative) renoprotection compared to the effects of BM-MSCs alone or the current standard of care medication, perindopril. Importantly, these broad renoprotective effects of BM-MSCs-eRLX + GFP were maintained in the presence of perindopril treatment, and even up to 9-months post-treatment cessation, suggesting that these combination cell-based treatments could effectively maintain their efficacy as adjunct therapies to clinically-used ACEi in the absence of any adverse side-effects.

Our study unveiled several noteworthy findings that significantly add to the current understanding of the application of genetically-engineered MSC-based therapies for treating CKD. Strikingly, a single i.v injection of BM-MSCs-eRLX + GFP significantly attenuated the IRI-induced kidney inflammation, tubular epithelial injury, interstitial kidney fibrosis and glomerulosclerosis after 7-days. Furthermore, a once-weekly i.v injection of BM-MSCs-eRLX + GFP over 2-weeks, significantly attenuated or normalised the HS-induced SBP, vascular endothelial and tubular epithelial damage, kidney inflammation, tubulointerstitial kidney fibrosis and glomerulosclerosis over a 14-day period. Most importantly, in addition to ameliorating factors that contribute to aberrant ECM/collagen accumulation (such as BP, epithelial tubular damage, immune cell infiltration, pro-inflammatory cytokine expression, pro-fibrotic TGF-β1/pSmad2 activity and myofibroblast accumulation), the anti-fibrotic effects of BM-MSCs-eRLX + GFP was mediated via their promotion of collagen-degrading gelatinase activity. This would be expected to facilitate the degradation of established collagen, consistent with the reported collagen-degrading effects of RLX [45, 53], an effect that would not likely to be provoked by BM-MSCs engineered to express other factors [12–15] that would only attenuate kidney damage-induced ECM production and deposition. Furthermore, these renoprotective effects of BM-MSCs-eRLX + GFP also led to the ability of these cells to normalise the HS-induced renal functional impairment after 14-days of treatment. Hence, this study importantly showed that the lentiviral transduction of RLX to human BM-MSCs did not compromise the broad therapeutic effects of RLX [6, 16]. Based on these collective findings, and those from a separate study that genetically-engineered a murine skeletal myoblast cell line to express RLX [54], it is proposed that the presence of RLX both reduced the fibrotic environment into which BM-MSCs-eRLX + GFP were administered into, and increased the number of viable BM-MSCs that could then exert greater therapeutic and reparative effects over that of BM-MSCs-eGFP alone.

As confirmation of this, BM-MSCs-eGFP alone failed to affect the IRI- or HS-induced inflammatory cell infiltration, tubular damage, glomerulosclerosis or interstitial myofibroblast accumulation when administered over the corresponding treatment period as BM-MSCs-eRLX + GFP in each model. Furthermore, despite exerting anti-hypertensive properties [28, 41] that have been shown to be mediated via the suppression of inflammatory factors that contribute to BP [41] or through their suppression of renin-angiotensin system components (angiotensin II, ACE, AT1 receptor) [55], BM-MSCs-eGFP alone did not ameliorate the HS-induced SBP to the same extent as BM-MSCs-eRLX + GFP. Similarly, the combined effects of BM-MSCs and RLX provided broader renoprotection over a current standard of care treatment for hypertensive CKD, perindopril, in HS-fed mice, which failed to affect the HS-induced inflammatory cell infiltration, tubular damage, glomerulosclerosis, gelatinase activity or creatinine clearance. These findings are consistent with previous studies that demonstrated the more rapid and broader renoprotective effects of BM-MSCs and RLX when combined, compared to perindopril in 1 K/DOCA/salt-hypertensive mice [28]. Given the slow-acting anti-fibrotic efficacy of ACEi [27, 56, 57], which induce anti-hypertensive and anti-inflammatory effects but do not resolve existing fibrosis, it is proposed that BM-MSCs-eRLX + GFP could even be employed as a stand-alone therapy for CKD, which induced similar anti-hypertensive effects to that of ACEi but broader renoprotection and rapid restoration of kidney functional impairment over these currently-used therapies.

It was also noted that a single i.v injection of BM-MSCs-eRLX + GFP abolished the HS-induced tubulointerstitial kidney fibrosis, glomerulosclerosis and kidney functional impairment, and stimulated gelatinase activity beyond that measured in NDW-fed mice by 14-days post-administration, to an equivalent extent as once-weekly administration of BM-MSCs-eRLX + GFP over the 2-week treatment period evaluated. This was despite BM-MSCs-eRLX + GFP (× 1) treatment having no significant effects on the HS-induced increase in SBP or tubular damage. These findings corroborated our previous studies in preclinical models of kidney [28, 39] and lung [58–60] disease, which indicated that the targeting of injury-induced fibrosis progression is likely to have a larger impact on alleviating kidney dysfunction compared to other pathological features of disease progression. Indeed, it has been determined that fibrosis is a major contributor to proteinuria [61], which may explain why all treatments evaluated that displayed anti-fibrotic effects, including perindopril which induced modest anti-fibrotic efficacy in HS-fed mice, were able to blunt or significantly reduce the HS-induced proteinuria. On the other hand, the regulation of creatinine clearance, which can be used to estimate GFR, requires the targeting of several disease-related pathologies [62], which perindopril failed to address in the current study. Hence, these findings highlighted the added benefit of engineering BM-MSCs with the anti-fibrotic properties of RLX.

## Conclusions

In summary, this study has showcased a revolutionary strategy for genetically engineering MSCs with an anti-fibrotic agent that promotes the MMP-induced resolution of established ECM. In doing so, it has highlighted the therapeutic potential of RLX-producing BM-MSCs as a novel treatment option for AKI and CKD. These engineered cells retained the characteristics of their naïve counterparts, but in acquiring the added presence of RLX, displayed enhanced viability post-transplantation and effectively attenuated or normalised injury-induced kidney inflammation, tubular injury, fibrosis and functional impairment, particularly when administered once-weekly over a 2-week period. Not only do these engineered cells provide a more clinically-feasible means of delivering the therapeutic impact of this combination therapy to human CKD patients, as BM-MSCs [6, 8, 9] RLX [16] or BM-MSCs-eRLX + GFP have demonstrated an excellent safety profile, this bodes well for the fast-tracking of BM-MSCs-eRLX + GFP as a clinically-viable treatment option for CKD.

## Supplementary Information

Additional file 1.

## Data Availability

The data supporting the findings from this study are available within the manuscript and its Supplementary Information. Any remaining raw data are archived in a Monash University repository and will be available from the corresponding authors upon reasonable request. Some data may not be available because of privacy or ethical restrictions.

## Abbreviations

CKD: Chronic kidney disease
BM-MSCs: Bone marrow-derived mesenchymal stromal cells
RLX: Human relaxin-2
BM-MSCs-eGFP: BM-MSCs-expressing green fluorescent protein
BM-MSCs-eRLX + GFP: BM-MSCs-expressing human relaxin-2 + green fluorescent protein
IRI: Ischemia reperfusion injury (IRI)
HS: High salt
ECM: Extracellular matrix
MMP: Matrix metalloproteinase
ACE: Angiotensin converting enzyme
CFU-F: colony-forming unit-fibroblasts
CD: Cluster of differentiation
HLA-DR: Human leukocyte antigen DR isotype
α-MEM: α-Minimal essential media
FBS: Fetal bovine serum
P: Passage
LV: Lentivirus
EF1A: Eukaryotic translation elongation factor-1α
MOI: Multiplicity of infection
RXFP1: Relaxin Family Peptide Receptor 1
DAPI: 4′,6-diamidino-2-phenylindole
FABP4: Fatty acid binding protein 4
NDW: Normal drinking water
SBP: Systolic blood pressure
gDNA: Genomic DNA
PCR: Polymerase chain reaction
OCT: Optimal cutting temperature
KIM: Kidney injury molecule
SMA: Smooth muscle actin
IgG: Immunoglobulin G
FACS: Fluorescence-activated cell sorting
ELISA: Enzyme-linked immunosorbent assay
MCP: Monocyte chemoattractant protein
TNF: Tumour necrosis factor
IL: Interleukin
TGF: Transforming growth factor
OD: Optical density
IGFBP: Insulin-like growth factor binding protein
uPAR: Urokinase-type plasminogen activation receptor
PDGF-AA: platelet-derived growth factor containing two A subunits
VEGF: Vascular endothelial growth factor
Smad2: Mothers against decapentaplegic homolog 2
