# Supplementary material for: The renoprotective efficacy and safety of genetically-engineered human bone marrow-derived mesenchymal stromal cells expressing anti-fibrotic cargo
Source: Stem Cell Res Ther. 2024 Oct 23;15:375. doi: 10.1186/s13287-024-03992-x (PMC11515549; doi:10.1186/s13287-024-03992-x)
Supplement: Supplementary file 1 — Additional file 1. [file 13287_2024_3992_MOESM1_ESM.docx]

**The renoprotective efficacy and safety of genetically-engineered human bone marrow-derived mesenchymal stromal cells expressing anti-fibrotic cargo**

Yifang Li^1,3^, Alex Hunter^1,3^, Miqdad M. Wakeel^1,3^, Guizhi Sun^2,4^, Ricky W.K. Lau^2,3^, Brad R.S. Broughton^1,3^, Ivan E. Oyarce Pino^1,3^, Zihao Deng^5^, Tingfang Zhang^2,3^, Padma Murthi^1,3^, Mark P. Del Borgo^1,3^, Robert E. Widdop^1,3^, Jose M. Polo^2,4,6,7^, Sharon D. Ricardo^2,3*^ & Chrishan S. Samuel^1,2,3,8*^

^1^Cardiovascular Disease Program and ^2^Development and Stem Cells Program, Monash Biomedicine Discovery Institute, Monash University, Clayton, Victoria 3800, Australia. ^3^Department of Pharmacology and ^4^Department of Anatomy and Developmental Biology, Monash University, Clayton, Victoria 3800, Australia.

^5^Department of Medicine (Alfred Hospital), Central Clinical School, Monash University, Melbourne, Victoria 3004, Australia.

^6^Adelaide Centre for Epigenetics, School of Biomedicine and ^7^The South Australian Immunogenomics Cancer Institute, The University of Adelaide, South Australia 5005, Australia.

^8^Department of Biochemistry and Pharmacology, The University of Melbourne, Parkville 3010, Victoria, Australia.

***Correspondence:** Professor Chrishan S. Samuel, PhD

Tel: +61 3 9902 0152 / E-mail: [chrishan.samuel@monash.edu](mailto:chrishan.samuel@monash.edu)

Professor Sharon D. Ricardo, PhD

E-mail: [sharon.ricardo@monash.edu](mailto:sharon.ricardo@monash.edu)

Department of Pharmacology, Monash University, Clayton, Victoria 3800, Australia

**Supplementary Table 1** The relative cytokine/chemokine intensity from the Proteome Profiler Human XL Cytokine Array analysis of naïve BM-MSCs vs BM-MSCs-eRLX+GFP

|  | Naïve BM-MSC-  secreted cytokine intensity | BM-MSC-eRLX+GFP-  secreted cytokine intensity |
| --- | --- | --- |
| **Immunomodulatory** |  |  |
| MCP-3 | 0.055 + 0.010 | 0.079 + 0.014 |
| IL-8 | 0.323 + 0.071 | 0.610 + 0.077* |
| IL-17A | 0.156 + 0.014 | 0.201 + 0.030 |
| VCAM-1 | 0.577 + 0.062 | 0.662 + 0.065 |
| **Angiogenic** |  |  |
| Relaxin-2 | 0.050 + 0.020 | 2.319 + 0.456** |
| VEGF | 1.623 + 0.291 | 1.404 + 0.305 |
| Endoglin | 0.177 + 0.030 | 0.265 + 0.057 |
| Angiopoietin 1 | 0.132 + 0.050 | 1.449 + 0.312** |
| Angiopoietin 2 | 0.116 + 0.017 | 0.164 + 0.012 |
| Angiogenin | 0.518 + 0.076 | 0.664 + 0.045 |
| IGFPB-3 | 0.913 + 0.069 | 0.817 + 0.091 |
| IGFPB-2 | 0.295 + 0.035 | 0.130 + 0.018* |
| **ECM remodelling and cell proliferative** |  |  |
| CD147 | 0.254 + 0.030 | 0.331 + 0.030 |
| Serpin E1 | 0.984 + 0.071 | 0.858 + 0.116 |
| uPAR | 0.274 + 0.036 | 0.227 + 0.086 |
| PDGF-AA | 0.123 + 0.018 | 0.088 + 0.010 |
| **Others detected** |  |  |
| FGF-19 | 0.178 + 0.009 | 0.198 + 0.011 |
| Thrombospondin-1 | 0.505 + 0.039 | 0.496 + 0.070 |
| SDF-1a | 0.276 + 0.021 | 0.299 + 0.020 |
| Osteopontin | 0.303 + 0.063 | 0.332 + 0.074 |
| GDF-15 | 0.606 + 0.061 | 0.655 + 0.066 |
| Cystatin C | 0.208 + 0.059 | 0.228 + 0.090 |
| IL-11 | 0.122 + 0.013 | 0.117 + 0.010 |

The mean ± SEM OD intensity of the cytokine/chemokines detected from naïve BM-MSCs vs BM-MSC-eRLX+GFP; from n=4 separate assays. *p<0.01, **p<0.01 vs corresponding levels from naïve BM-MSCs.

**Supplementary Table 2** List of antibodies used for flow cytometry

| Antigen | Host/Isotype | Clone | Tag | Dilution | Company |
| --- | --- | --- | --- | --- | --- |
| CD45 | Rat IgG2b, κ | 30-F11 | PECY5 | 1:200 | BioLegend,USA |
| CD11C | Armenian Hamster IgG1, λ2 | Clone HL3 | BUV395 | 1:100 | BD Biosciences, USA |
| F4/80 | Rat IgG2a, κ | BM8 | APC-Fire 750 | 1:100 | BioLegend, USA |
| CD206 | Rat IgG2a, κ | C068C2 | AF647 | 1:100 | BioLegend, USA |

**Supplementary Table 3** The effects of IRI or HS and the respective treatments investigated on animal body weight (BW), kidney weight (KW) and KW to BW ratio

| **Parameter measured** | **BW**  **(g)** | **Left KW**  **(mg)** | **LKW:**  **BW ratio**  **(mg/g)** | **Right KW**  **(mg)** | **RKW:**  **BW ratio (mg/g)** |
| --- | --- | --- | --- | --- | --- |
| Sham [8] | 29 ± 1 | 181 ± 4 | 6.3 ± 0.1 | 196 ± 4 | 6.8 ± 0.1 |
| IRI [8] | 28 ± 1 | 190 ± 5 | 6.8 ± 0.2 | 199 ± 7 | 7.2 ± 0.3 |
| IRI+BM-MSCs-eGFP [8] | 27 ± 1 | 190 ± 6 | 7.0 ± 0.2 | 202 ± 6 | 7.4 ± 0.2 |
| IRI+BM-MSCs-RLX+GFP [8] | 27 ± 1 | 188 ± 6 | 6.9 ± 0.2 | 187 ± 6 | 6.8 ± 0.3 |
| IRI+Pump RLX+BM-MSCs [8] | 28 ± 1 | 189 ± 4 | 6.7 ± 0.1 | 198 ± 5 | 7.0 ± 0.2 |
|  |  |  |  |  |  |
| NDW [8] | 35 ± 1 | 284 ± 9 | 8.3 ± 0.3 | 287 ± 9 | 8.4 ± 0.3 |
| HS [8] | 30 ± 1* | 273 ± 11 | 9.0 ± 0.3 | 272 ± 13 | 9.0 ± 0.4 |
| HS+BM-MSCs-eGFP (x2) [8] | 32 ± 1 | 290 ± 14 | 9.0 ± 0.3 | 290 ± 17 | 9.0 ± 0.4 |
| HS+BM-MSCs-eRLX+eGFP (x1) [7] | 32 ± 1 | 282 ± 11 | 8.8 ± 0.2 | 290 ± 14 | 9.1 ± 0.3 |
| HS+BM-MSCs-eRLX+eGFP (x2) [7] | 33 ± 1 | 268 ± 6 | 8.2 ± 0.1 | 254 ± 8 | 7.7 ± 0.2 |
| HS+Pump RLX+BM-MSCs [8] | 32 ± 1 | 265 ± 6 | 8.3 ± 0.2 | 271 ± 10 | 8.4 ± 0.3 |
| HS+Perindopril [8] | 32 ± 1 | 269 ± 19 | 8.1 ± 0.4 | 285 ± 19 | 8.6 ± 0.4 |

All data are expressed as the mean+SEM; from n=6-8 mice per group (as denoted by the numbers in the square brackets). *p<0.05 vs NDW control group.

**
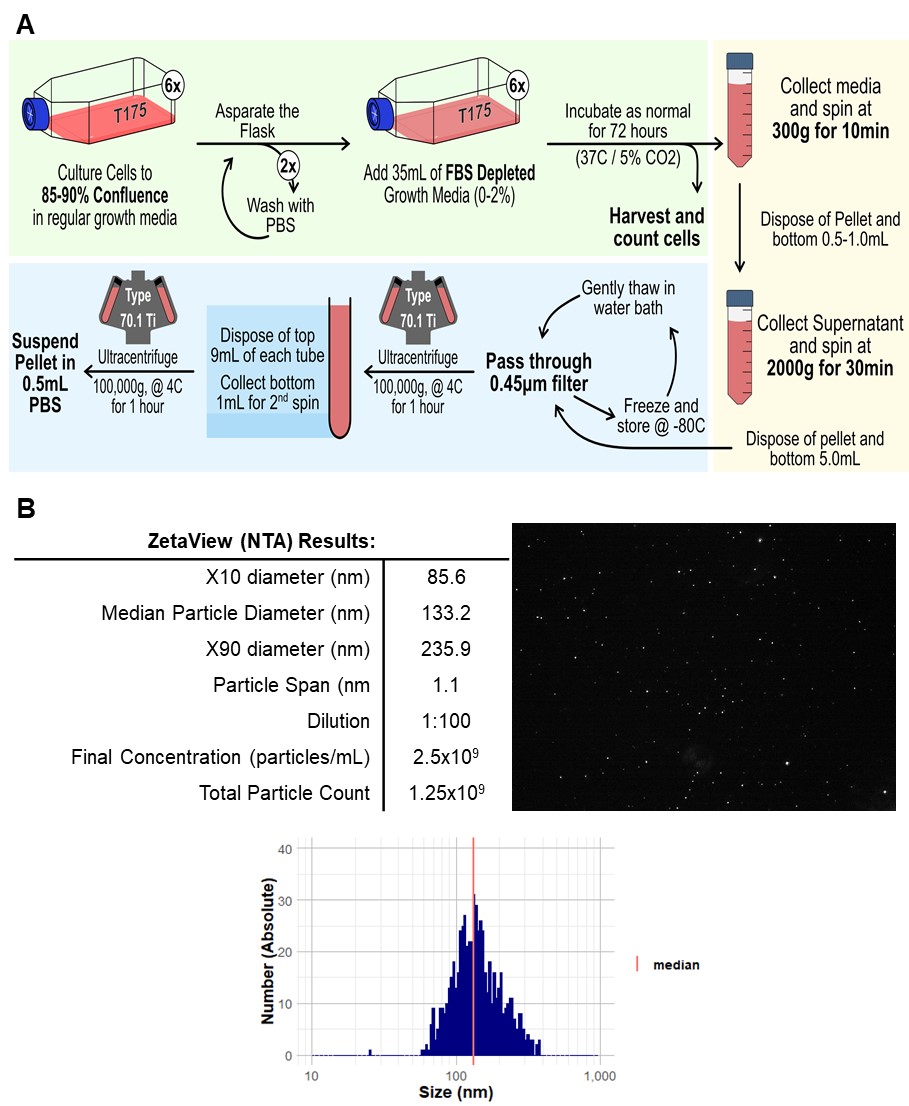
**

**Supplementary Fig. 1** Extraction and characterisation of BM-MSCs-eRLX+GFP-derived exosomes. (**A**) Shown is a flowchart outlining how exosomes were harvested and isolated from BM-MSCs-eRLX+GFP (completed once). (**B**) BM-MSCs-eRLX+GFP-derived exosomes were characterised using the Zetaview® PMX-120 Nanoparticle Tracking Analyzer (NTA), using a 1:100 dilution. A total of 1.25x10^9^ particles were isolated, with a median diameter of 133.2nm (X10 = 85.6nm, X90 = 235.9nm, span = 1.1).

**
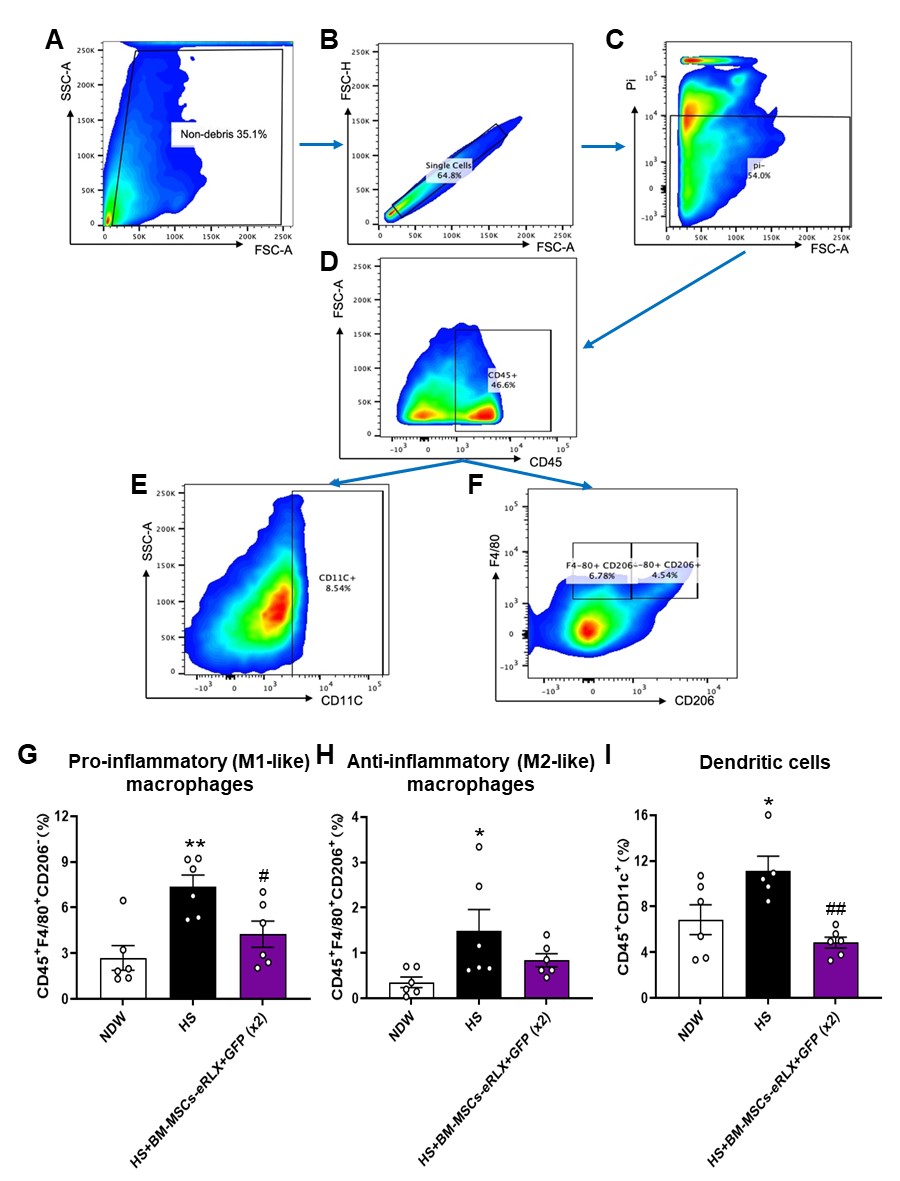
**

**Supplementary Fig. 2** Flow cytometric analysis of the effects of HS and BM-MSCs-eRLX+GFP on macrophage subsets and dendritic infiltration within the kidney. (**A**) Total non-cellular debris (including all monocytes, lymphocytes and granulocytes) was gated by forward scatter area (FSC-A) vs side scatter area (SSC-A). (**B**) Live leukocyte singlets were then gated by forward scatter-height (FSC-H) vs FSC-A, followed by (**C**) exclusion of dead cells (PI stain), and (**D**) gated as CD45^+^ populations against FSC-A. These leukocytes were then divided into (**E**) dendritic cells (CD45^+^CD11c^+^) or (**F**) CD45^+^F4/80^+^CD206^−^ pro-inflammatory (M1-like) macrophages and CD45^+^F4/80^+^CD206^+^ anti-inflammatory (M2-like) macrophages. (**G**) Also shown are the mean + SEM % CD45^+^F4/80^+^CD206^−^ pro-inflammatory (M1-like) macrophage, CD45^+^F4/80^+^CD206^+^ anti-inflammatory (M2-like) macrophage (H) and CD45^+^CD11c^+^ dendritic cell infiltration (I) in the kidneys of NDW controls (n=6), HS-fed mice (n=6) and HS-fed mice treated with BM-MSCs-eRLX+GFP (x2) (n=6), as determined by flow cytometry analysis. *p<0.05, **p<0.01 vs the NDW control group; ^#^p<0.05, ^##^p<0.01 vs the HS alone group; as determined using a one-way ANOVA and Tukey’s *post-hoc* test.

**
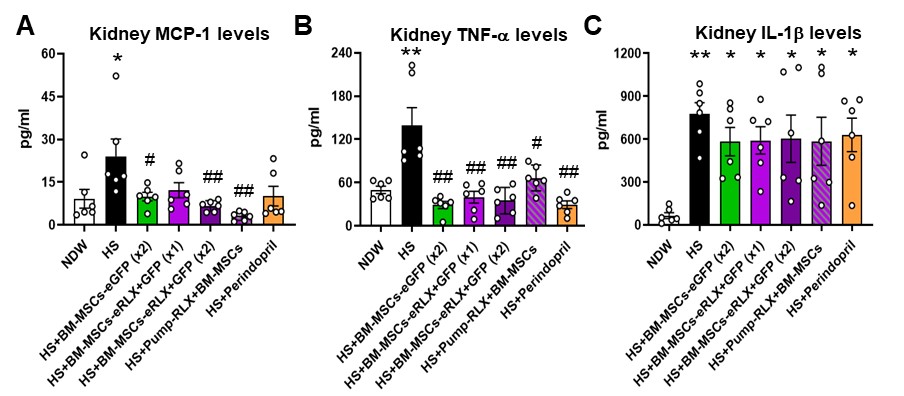
**

**Supplementary Fig. 3** The effects of HS and the treatments evaluated on pro-inflammatory cytokine expression levels within the kidney. (**A-C**) Shown are the mean ± SEM levels of (**A**) monocyte chemoattractant protein (MCP)-1, (**B**) tumour necrosis factor (TNF)-α and (**C**) interleukin (IL)-1β within the kidney, as determined by ELISA analysis of kidney protein extracts; from n=6 mice per group evaluated. *p<0.05, **p<0.01 vs the NDW control group; ^#^p<0.05, ^##^p<0.01 vs the HS alone group; as determined using a one-way ANOVA and Tukey’s *post-hoc* test.


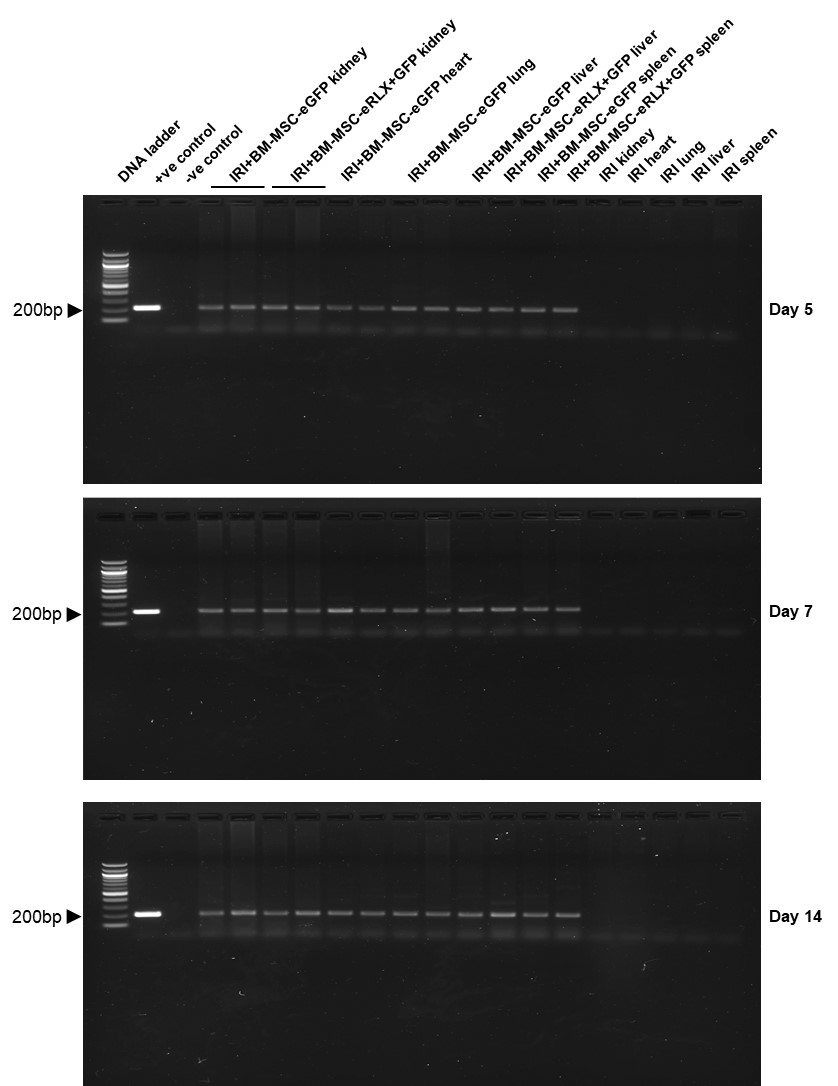


**Supplementary Fig. 4** The full-length agarose gel blots that were cropped to create Fig. 3A.


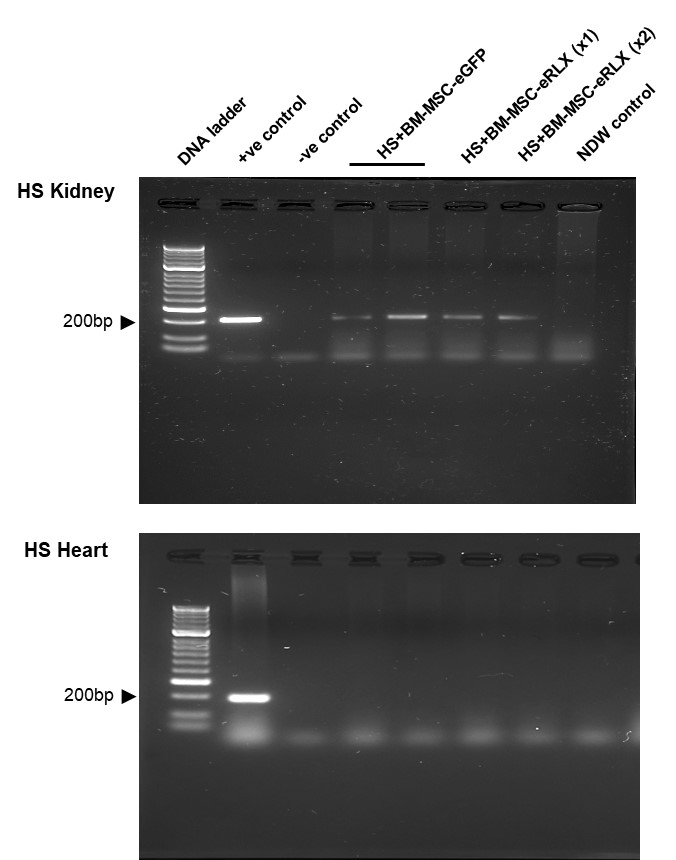


**Supplementary Fig. 5** The full-length agarose gel blots that were cropped to create Fig. 3D.
